# Dataset of pollen morphological traits of 56 dominant species among desert vegetation in the eastern arid central Asia

**DOI:** 10.1016/j.dib.2018.03.122

**Published:** 2018-03-31

**Authors:** Kai-Qing Lu, Gan Xie, Min Li, Jin-Feng Li, Anjali Trivedi, David K. Ferguson, Yi-Feng Yao, Yu-Fei Wang

**Affiliations:** a State Key Laboratory of Systematic and Evolutionary Botany, Institute of Botany, Chinese Academy of Sciences, 20 Nanxincun Xiangshan, Beijing 100093, China; b Birbal Sahni Institute of Palaeosciences, 53 University Road, Lucknow 226007, India; c University of Vienna, Institute of Palaeontology, Althanstrasse 14, Vienna A-1090, Austria; d University of Chinese Academy of Sciences, Beijing 100039, China

**Keywords:** Arid central Asia, Desert vegetation, Pollen morphological traits, Temperate desert

## Abstract

The data presented in this article are related to the research article entitled “Pollen spectrum, a cornerstone for tracing the evolution of the eastern central Asian desert” (JQSR 5260) (Lu et al., 2018) [1] In this paper, we supply a dataset, which provides a descriptive and general summary of pollen characteristic of desert dominant species in the eastern arid central Asia (ACA). The other important component is the illustration on pollen grains traits under light microscopy (LM) and scanning electron microscopy (SEM). Pollen grains of 56 species are extracted from voucher specimens from the PE herbarium at the Institute of Botany. It is worth noting that these species own special distribution patterns in China. The distribution maps are plotted using the Google Maps and the species distribution data at the county level supplied by the Chinese Virtual Herbarium (http://www.cvh.ac.cn/).

**Specifications Table**

**Table:** 

Subject area	*Botany*
More specific subject area	*Palynology and morphology of desert dominant plant species.*
Type of data	*Tables of the voucher specimen list and pollen morphological characters*
	*Illustrations of microscope images for pollen grains*
	*Distribution maps for these species*
How data was acquired	*Microscope, SEM*
	*Field investigations and collections*
Data format	Tables in MS Word format *.doc
	*Illustrations of microscope images in 32-bit RGB JPG (300dpi)*
	*Distribution maps in 32-bit RGB JPG (300dpi)*
Experimental factors	*Pollen grains are acetolyzed by the standard method and fixed in glycerin jelly.*
Experimental features	*Standard procedures are followed for light microscopy and scanning electron microscopy.*
Data source location	*China*
Data accessibility	*The data are available with this article*
Related research article	*K.Q. Lu, G. Xie, M. Li, J.F. Li, A. Trivedi, D. K. Ferguson, Y.F. Yao, Y.F. Wang, Pollen spectrum, a cornerstone for tracing the evolution of the eastern central Asian desert, Quat. Sci. Rev. 186, 2018, 111-122.*

**Value of the Data**

• The dataset includes pollen morphological characteristics and distribution patterns of 56 dominant species among the desert vegetation in eastern ACA.
• The pollen descriptions with related illustrations could be used to identify pollen grains at the generic or species level.
• The distribution maps could be used to interprete distribution patterns of 56 dominant species among the desert vegetation in eastern ACA.

## 1 Data

The dataset of this article provides information on the diversity of pollen features of the dominant species in the eastern ACA desert and the distribution patterns of these species in China. Plate 1, Plate 2, Plate 3, Plate 4, Plate 5, Plate 6, Plate 7, Plate 8, Plate 9, Plate 10, Plate 11 and Table 1 show the diversity of pollen morphology. Fig. 1, Fig. 2, Fig. 3, Fig. 4, Fig. 5, Fig. 6, Fig. 7 present the distribution patterns of the 56 species in China. The pollen descriptions are provided in Appendix A.

### 1.1 Pollen images of the 56 dominant species in the eastern arid Central Asia desert

See plates Plate 1, Plate 2, Plate 3, Plate 4, Plate 5, Plate 6, Plate 7, Plate 8, Plate 9, Plate 10, Plate 11.

**Plate 1:**
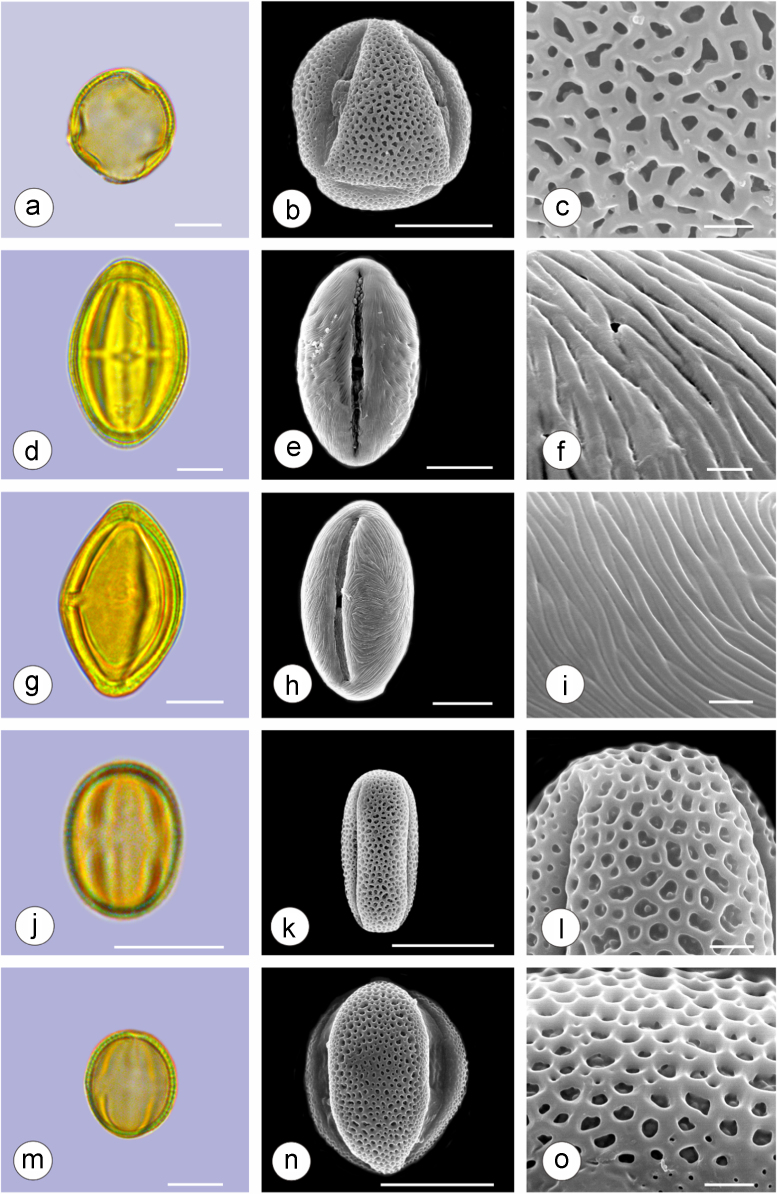
a-c. *Peganum harmala;* d-f. *Nitraria roborowskii*; g-i. *Nitraria sphaerocarpa*; j-l. *Tetraena mongolica*; m-o. *Zygophyllum xanthoxylon*. Bar in LM and SEM overview 10 μm, in SEM close-up 1 μm.

**Plate 2:**
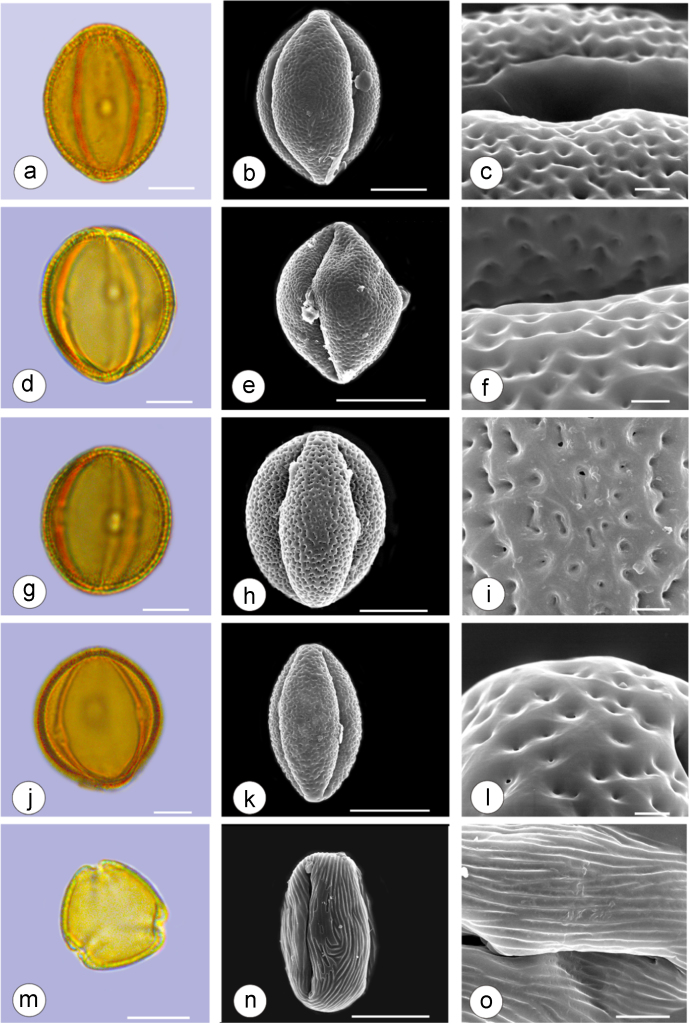
a-c. *Calligonum roborowskii*; d-f. *Calligonum mongolicum*; g-i. *Calligonum leucocladum*; j-l. *Calligonum rubicundum*; m-o. *Potaninia mongolica*. Bar in LM and SEM overview 10 μm, in SEM close-up 1 μm.

**Plate 3:**
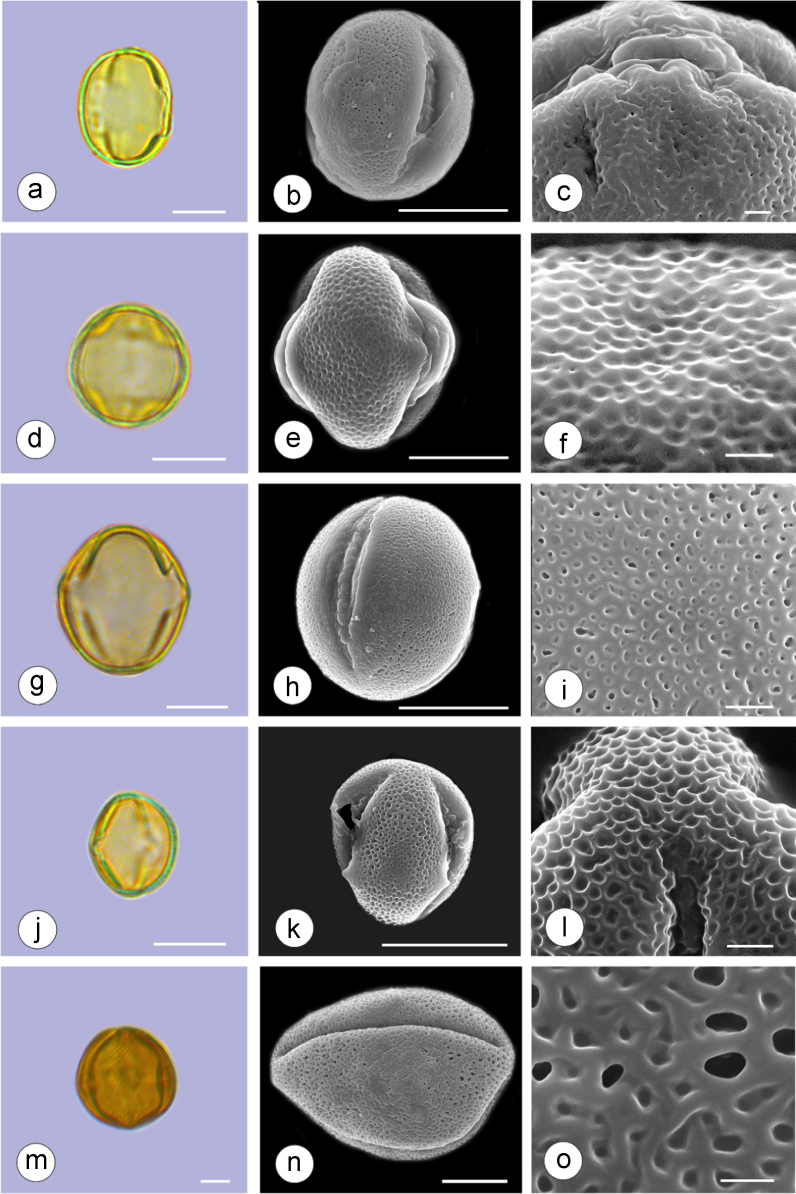
a-c. *Ammopiptanthus mongolicus*; d-f. *Ammodendron bifolium*; g-i. *Caragana korshinskii*; j-l. *Alhagi sparsifolia*; m-o. *Helianthemum songaricum*. Bar in LM and SEM overview 10 μm, in SEM close-up 1 μm.

**Plate 4:**
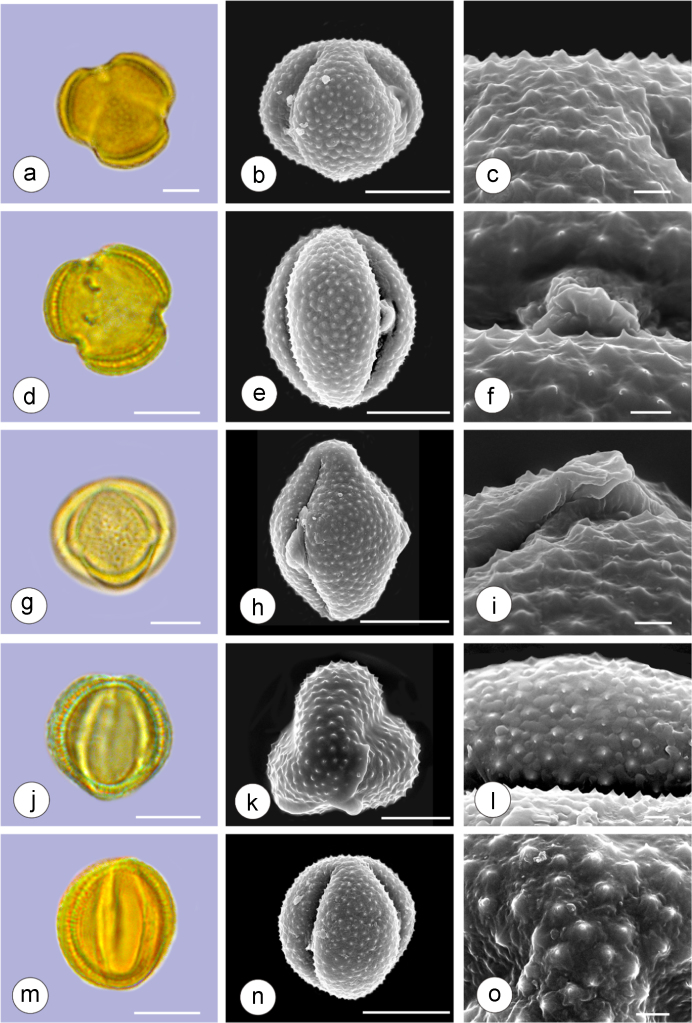
a-c. *Seriphidium santolinum*; d-f. *Seriphidium borotalense*; g-i. *Seriphidium rhodanthum*; j-l. *Seriphidium kaschgaricum*; m-o. *Seriphidium terrae-albae*. Bar in LM and SEM overview 10 μm, in SEM close-up 1 μm.

**Plate 5:**
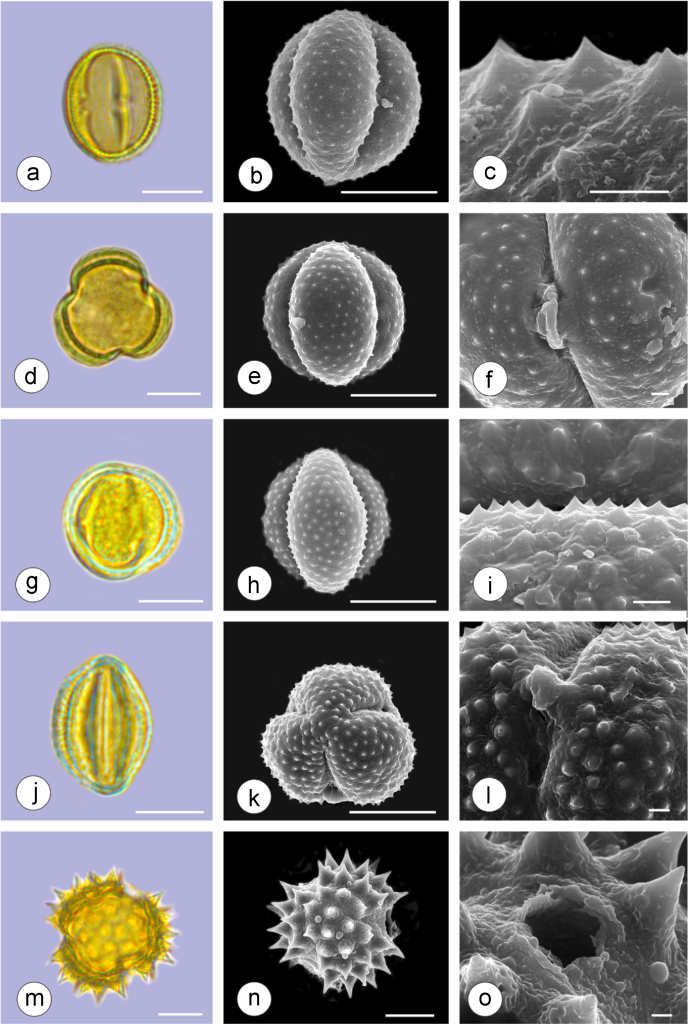
a-c. *Artemisia ordosica*; d-f. *Artemisia sphaerocephala*; g-i. *Artemisia nanschanica*; j-l. *Artemisia desertorum*; m-o. *Karelinia caspia*. Bar in LM and SEM overview 10 μm, in SEM close-up 1 μm.

**Plate 6:**
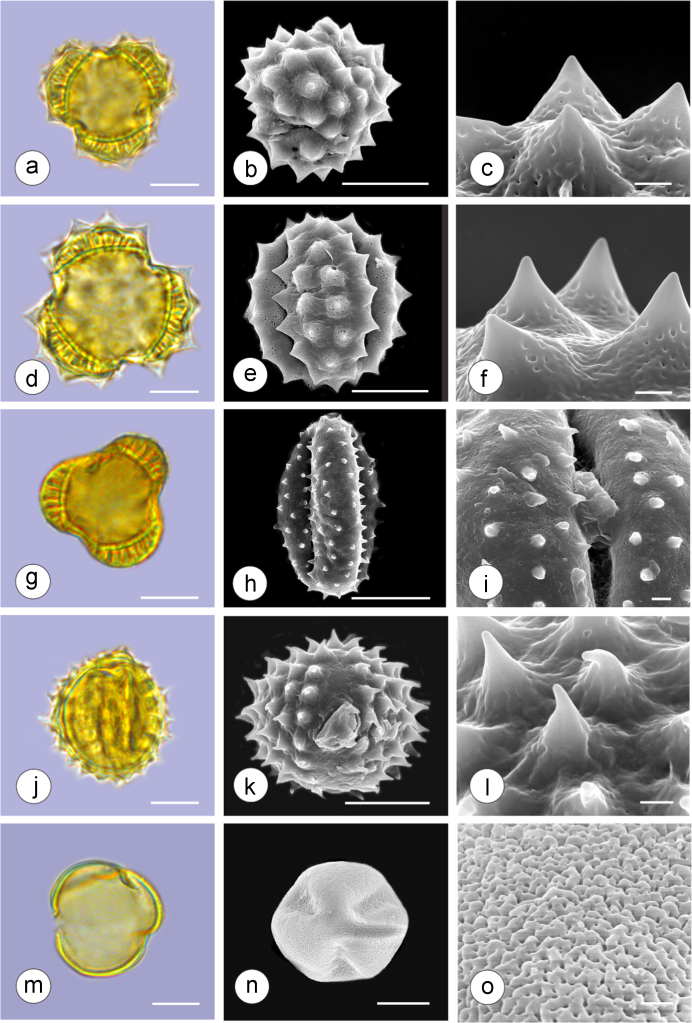
a-c. *Ajania fruticulosa*; d-f. *Ajania fastigiata*; g-i. *Ajania tibetica*; j-l. *Asterothamnus centrali-asiaticus*; m-o. *Cistanche deserticola*. Bar in LM and SEM overview 10 μm, in SEM close-up 1 μm.

**Plate 7:**
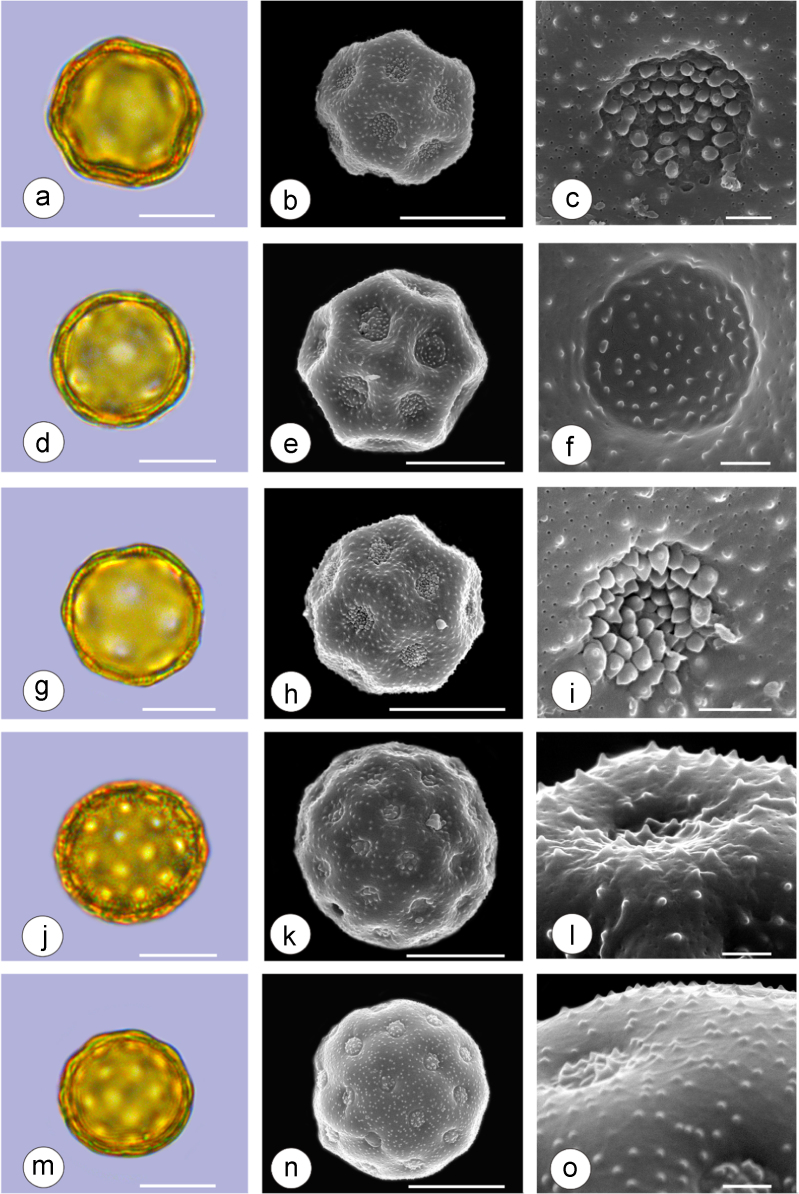
a-c. *Anabasis aphylla*; d-f. *Anabasis salsa*; g-i. *Anabasis brevifolia*; j-l. *Atriplex cana*; m-o. *Halostachys caspica*. Bar in LM and SEM overview 10 μm, in SEM close-up 1 μm.

**Plate 8:**
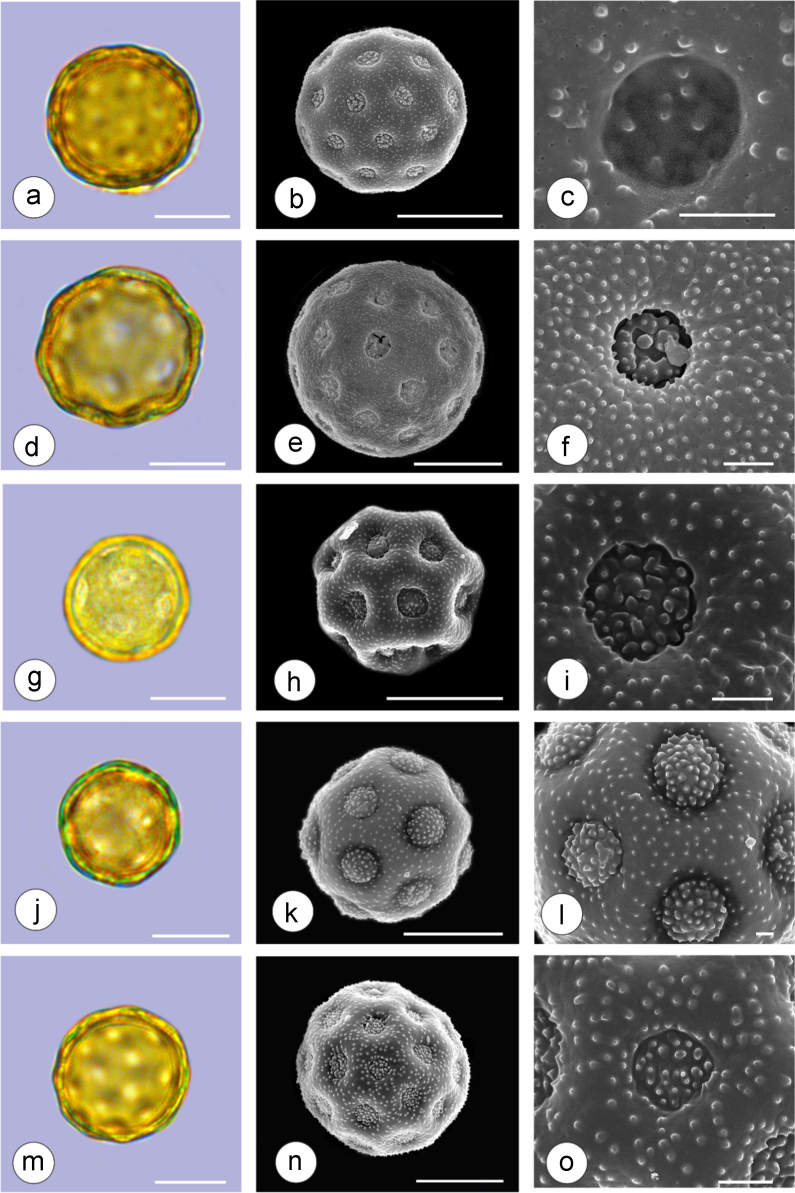
a-c. *Halocnemum strobilaceum*; d-f. *Haloxylon ammodendron*; g-i. *Haloxylon persicum*; j-l. *Iljinia regelii*; m-o. *Sympegma regelii*. Bar in LM and SEM overview 10 μm, in SEM close-up 1 μm.

**Plate 9:**
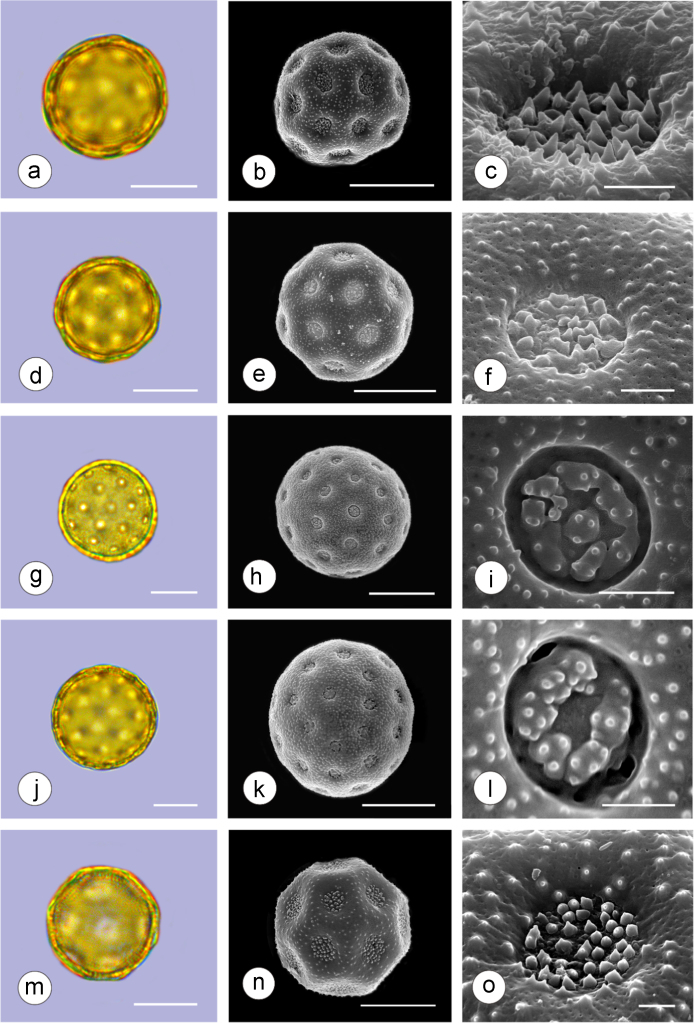
a-c. *Kalidium schrenkianum*; d-f. *Kalidium cuspidatum*; g-i. *Krascheninnikovia compacta*; j-l. *Krascheninnikovia ceratoides*; m-o. *Nanophyton erinaceum*. Bar in LM and SEM overview 10 μm, in SEM close-up 1 μm.

**Plate 10:**
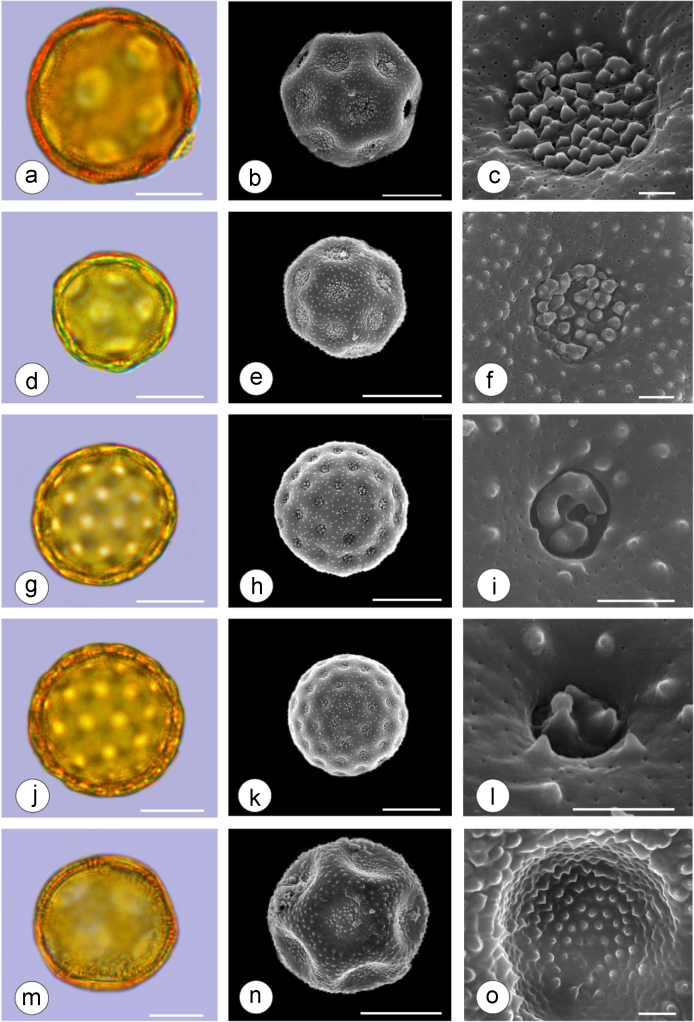
a-c. *Salsola passerina*; d-f. *Salsola abrotanoides*; g-i. *Suaeda physophora*; j-l. *Suaeda microphylla*; m-o. *Gymnocarpos przewalskii*. Bar in LM and SEM overview 10 μm, in SEM close-up 1 μm.

**Plate 11:**
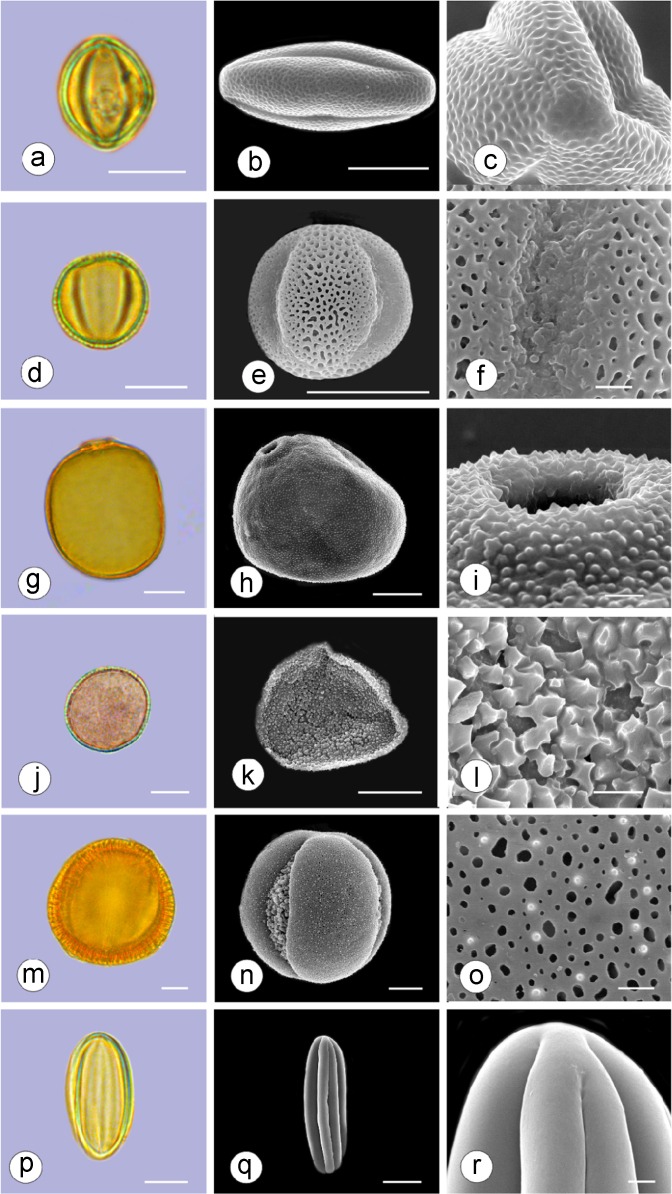
a-c. *Reaumuria soongarica*; d-f. *Tamarix chinensis*; g-i. *Psammochloa villosa*; j-l. *Populus euphratica*; m-o. *Convolvulus tragacanthoides*; p-r. *Ephedra przewalskii*. Bar in LM and SEM overview 10 μm, in SEM close-up 1 μm.

### 1.2 Data of pollen morphological characteristics

See Table 1.

**Table 1:** Pollen morphological characters of 56 dominant and indicative species in NW China, eastern ACA.

**Species**	**Polar axis /μm**	**Equatorial axis /μm**	**P/E (mean)**	**Shape**	**Aperture**	**Exine ornamentation**	**Exine thickness /μm**	**Plates**
*Peganum harmala*	(21.5-)23.5 (-25.1)	(19.0-)21.5 (-23.3)	1.09	spheroidal	tricolporate	reticulate	1.7	1a-c
*Nitraria roborowskii*	(31.4-)34.5 (-36.8)	(19.6-)22.0 (-24.2)	1.57	prolate	tricolporate	striate	2.7	1d-f
*Nitraria sphaerocarpa*	(28.8-) 33.9 (-38.5)	(21.8-)23.8 (-26.7)	1.42	prolate	tricolporate	striate	2.5	1g-i
*Tetraena mongolica*	(13.1-)15.0(-16.3)	(11.5-)12.6 (-13.7)	1.19	subprolate	tricolporate	reticulate	1.1	1j-l
*Zygophyllum xanthoxylon*	(16.3-)19.0(-23.2)	(13.8-)16.5 (-20.)	1.16	subprolate	tricolporate	reticulate	1.6	1m-o
*Calligonum roborowskii*	(30.1-)32.3 (-37.8)	(25.5-)28.3 (-32.8)	1.05	spheroidal	tricolporate	foveolate	2.3	2a-c
*Calligonum mongolicum*	(34.5-)35.9 (-38.6)	(32.4-)34.1 (-35.4)	1.09	spheroidal	tricolporate	foveolate	2.6	2d-f
*Calligonum leucocladum*	(26.7-)30.4 (-32.1)	(22.8-)27.4 (-30.5)	1.11	spheroidal	tricolporate	foveolate	2.4	2g-i
*Calligonum rubicundum*	(26.9-)33.5 (-40.2)	(23.9-)29.7 (-35.9)	1.13	spheroidal	tricolporate	foveolate	2.3	2j-l
*Potaninia mongolica*	(16.3-)20.3 (-25.4)	(13.3-)17.9 (-20.8)	1.14	subprolate	tricolporate	striate	1.7	2m-o
*Ammopiptanthus mongolicus*	(15.3-)21.1 (-23.3)	(13.3-)16.9 (-18.4)	1.25	subprolate	tricolporate	microreticulate	1.3	3a-c
*Ammodendron bifolium*	(15.9-)17.9 (-20.4)	(14.9-)17.1 (-18.9)	1.05	spheroidal	tricolporate	microreticulate	1.4	3d-f
*Caragana korshinskii*	(17.8-) 21.3 (-25.5)	(13.3-)17.4 (-21.9)	1.24	subprolate	tricolporate	foveolate	1.6	3g-i
*Alhagi sparsifolia*	(13.1-)13.7 (-17.1)	(11.9-)13.7 (-15.3)	1.08	spheroidal	tricolporate	microreticulate	1.2	3j-l
*Helianthemum songaricum*	(37.8-) 39.6 (-40.4)	(33.7-)37.6 (-39.8)	1.05	spheroidal	tricolporate	reticulate	3.9	3m-o
*Seriphidium santolinum*	(20.5-)22.7 (-25.1)	(20.9-)23.1 (-26.1)	0.98	spheroidal	tricolporate	microechinate	3.0	4a-c
*Seriphidium borotalense*	(17.4-)19.6 (-22.7)	(14.6-)19.4 (-21.2)	1.02	spheroidal	tricolporate	microechinate	2.8	4d -f
*Seriphidium rhodanthum*	(19.6-)22.5 (-29.5)	(20.4-)22 (24.2)	1.02	spheroidal	tricolporate	microechinate	2.6	4g-i
*Seriphidium kaschgaricum*	(20.4-)21.9 (-24.5)	(19.5-)21.3 (-22.6)	1.03	spheroidal	tricolporate	microechinate	3.1	4j-l
*Seriphidium terrae-albae*	(18.0-)21.4 (-26.1)	(17.7-)21.1 (-27.0)	1.01	spheroidal	tricolporate	microechinate	2.8	4m-o
*Artemisia ordosica*	(17.6-)20.5 (-23.5)	(17.0-)18.3 (-21.3)	1.12	spheroidal	tricolporate	microechinate	2.4	5a-c
*Artemisia sphaerocephala*	(17.6-)23.2 (-25.3)	(16.0-) 21 (-23.9)	1.11	spheroidal	tricolporate	microechinate	3.0	5d-f
*Artemisia nanschanica*	(17.5-)20.2 (-24.4)	(15.3-)18.1 (-20.1)	1.12	spheroidal	tricolporate	microechinate	2.5	5g-i
*Artemisia desertorum*	(16.7-)20.9 (-24.7)	(14.6-)19.0 (-21.0)	1.10	spheroidal	tricolporate	microechinate	2.7	5j-l
*Karelinia caspia*	(18.7-)25.0 (-29.6)	(19.8-)23.7 (-26.7)	1.07	spheroidal	tricolporate	echinate-perforate	3.3	5m-o
*Ajania fruticulosa*	(22.7-)24.4 (-26.4)	(21.2-)23.3 (-26.2)	1.05	spheroidal	tricolporate	echinate-perforate	3.6	6a-c
*Ajania fastigiata*	(22.2-)27.0 (-31.9)	(18.6-)25.3 (-31.9)	1.07	spheroidal	tricolporate	echinate-perforate	3.8	6d-f
*Ajania tibetica*	(21.0-)23.2 (-26.7)	(20.3-)22.2 (-26.0)	1.05	spheroidal	tricolporate	echinate	3.3	6g-i
*Asterothamnus centrali-asiaticus*	(25.5-)28.2 (-36.0)	(24.0-)26.9 (-31.3)	1.05	spheroidal	tricolporate	echinate-perforate	2.6	6j-l
*Cistanche deserticola*	(24.7-)28.5 (-34.1)	(16.3-)23.9 (-28.1)	1.21	subprolate	tricolpate	reticulate	1.8	6m-o
*Anabasis aphylla*	(14.9-)17.7 (-20.9)	(14.6-)16.9 (-20.2)	1.05	spheroidal	pantoporate	Microechinate- perforate	2.0	7a-c
*Anabasis salsa*	(13.8-)18.4 (-24.8)	(13.3-)17.9 (-22.9)	1.03	spheroidal	pantoporate	microechinate- perforate	1.9	7d-f
*Anabasis brevifolia*	(16.2-)19.2 (-20.9)	(14.9-)18.3 (-20.7)	1.05	spheroidal	pantoporate	microechinate- perforate	2.0	7g-i
*Atriplex cana*	(16.3-)19.2 (-22.2)	(14.8-)18.6 (-21.2)	1.04	spheroidal	pantoporate	microechinate- perforate	2.1	7j-l
*Halostachys caspica*	(14.7-)18 (-20.9)	(14.5-)16.9 (-20.6)	1.06	spheroidal	pantoporate	microechinate- perforate	1.8	7m-o
*Halocnemum strobilaceum*	(14.8-)17.3 (-18.8)	(13.5-)16.5 (-18.4)	1.05	spheroidal	pantoporate	microechinate- perforate	1.9	8a-c
*Haloxylon ammodendron*	(15.9-)19.2 (-21.6)	(15.3-)18.8 (-21.4)	1.02	spheroidal	pantoporate	microechinate- perforate	1.4	8d-f
*Haloxylon persicum*	(11.7-)16.6 (-19.3)	(11.4-) 16.1(18.9)	1.03	spheroidal	pantoporate	microechinate- perforate	1.6	8g-i
*Iljinia regelii*	(11.1-)12.5 (-13.9)	(10.7-)12.1 (-13.4)	1.03	spheroidal	pantoporate	microechinate- perforate	1.4	8j-l
*Sympegma regelii*	(15.7-)18.5 (-21.9)	(15.1-)17.6 (-21.4)	1.05	spheroidal	pantoporate	microechinate- perforate	1.8	8m-o
*Kalidium schrenkianum*	(16.7-)19 (-22.1)	(15.8-)18.2 (-21.5)	1.05	spheroidal	pantoporate	microechinate- perforate	2.0	9a-c
*Kalidium cuspidatum*	(14.1-)17.4 (-19.6)	(12.6-)16.5 (-18.8)	1.05	spheroidal	pantoporate	microechinate- perforate	1.8	9d-f
*Krascheninnikovia compacta*	(20.3-)23.8 (-27.2)	(20.4-)22.6 (-25)	1.05	spheroidal	pantoporate	microechinate- perforate	2.3	9g-i
*Krascheninnikovia ceratoides*	(19.2-)24.6 (-25.7)	(18.6-)23.4 (-25.5)	1.05	spheroidal	pantoporate	microechinate- perforate	2.2	9j-l
*Nanophyton erinaceum*	(16.4-)18.6 (-19.8)	(16.0-)18.0 (-19.2)	1.03	spheroidal	pantoporate	microechinate- perforate	1.8	9m-o
*Salsola passerina*	(21.0-)24.9 (-27.2)	(20.7-)24.2 (-26.7)	1.03	spheroidal	pantoporate	microechinate- perforate	2.3	10a-c
*Salsola abrotanoides*	(17.2-)19.0 (-20.8)	(16.9-)18.3 (-20.1)	1.04	spheroidal	pantoporate	microechinate- perforate	1.9	10d-f
*Suaeda physophora*	(17.3-)18.8 (-21.8)	(14.8-)17.5 (-20.6)	1.07	spheroidal	pantoporate	microechinate- perforate	1.9	10g-i
*Suaeda microphylla*	(20.0-)23.3 (-27.5)	(17.7-)22.4 (-26.7)	1.04	spheroidal	pantoporate	microechinate- perforate	2.2	10j-l
*Gymnocarpos przewalskii*	(18.4-)23.6 (-27.8)	(17.1-)22.7 (-26.5)	1.04	spheroidal	pantoporate	microechinate- perforate	2.2	10m-o
*Reaumuria soongarica*	(11.5-)13.1 (-14.9)	(9.0-)10.4 (-12.1)	1.27	subprolate	tricolpate	reticulate	1.2	11a-c
*Tamarix chinensis*	(13.2-)16.3 (-20.7)	(14.0-)15.5 (-18.4)	1.14	spheroidal	tricolpate	reticulate	1.7	11d-f
*Psammochloa villosa*	(40.9-)43.1 (-46.8)	(31.3-)35.2 (-42.5)	1.23	subprolate	monoporate	microgranulate	2.3	11g-i
*Populus euphratica*	(19.3-)26.8 (-37.2)	(19.1-)25.0 (-36.4)	1.07	spheroidal	inaperturate	granulate	1.6	11j-l
*Convolvulus tragacanthoides*	(51.7-) 61.9 (-78.4)	(34.5-)47.5 (-63.8)	1.32	subprolate	tricolpate	reticulate- granulate	5.5	11m-o
*Ephedra przewalskii*	(24.5-)29.9 (-34.2)	(14.8-)16.3 (-18.2)	1.84	prolate	inaperturate	fossulate	2.0	11p-r

### 1.3 Distribution maps of the 56 dominant species in China

**Fig. 1:**
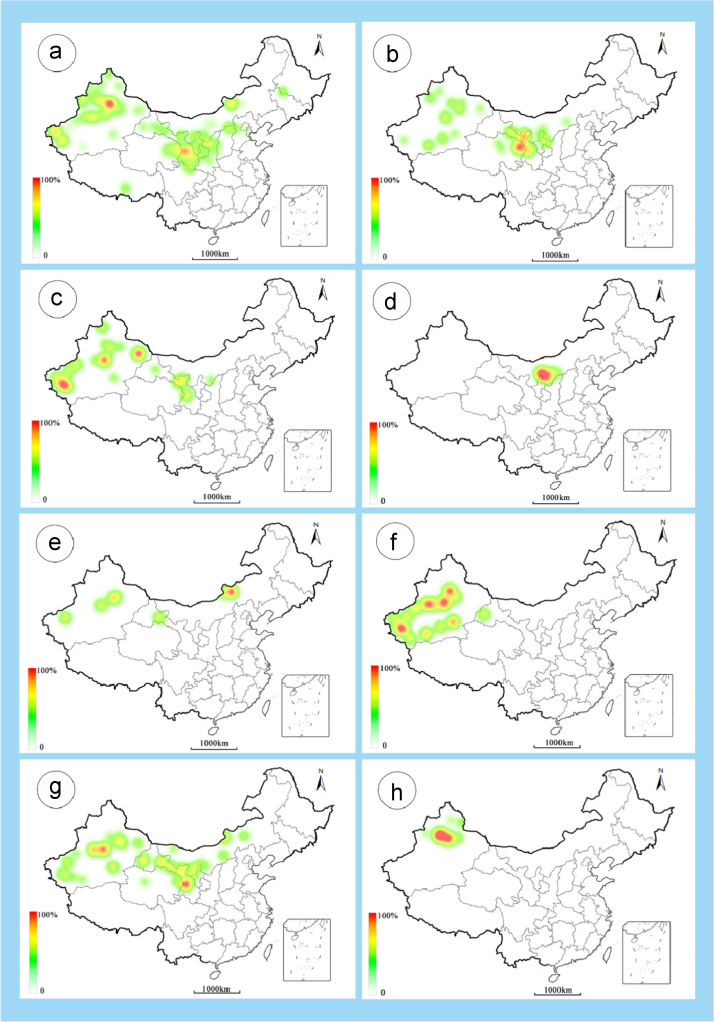
a. *Peganum harmala*; b. *Nitraria roborowskii*; c. *Nitraria sphaerocarpa*; d. *Tetraena mongolica*; e. *Zygophyllum xanthoxylon*; f. *Calligonum roborowskii*; g. *Calligonum mongolicum*; h. *Calligonum leucocladum*. The colour of the bars from red to white representing the probability of the species occurrence decreasing.

**Fig. 2:**
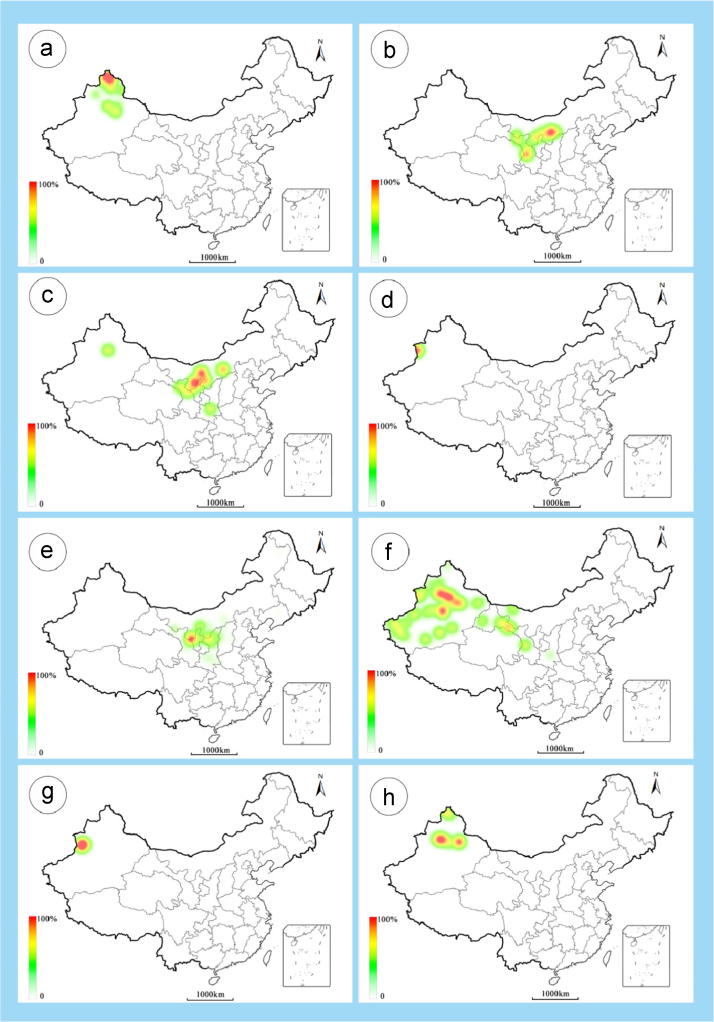
a. *Calligonum rubicundum*; b. *Potaninia mongolica*. c. *Ammopiptanthus mongolicus*; d. *Ammodendron bifolium*; e. *Caragana korshinskii*; f. *Alhagi sparsifolia*; g. *Helianthemum songaricum*; h. *Seriphidium santolinum*. The colour of the bars from red to white representing the probability of the species occurrence decreasing.

**Fig. 3:**
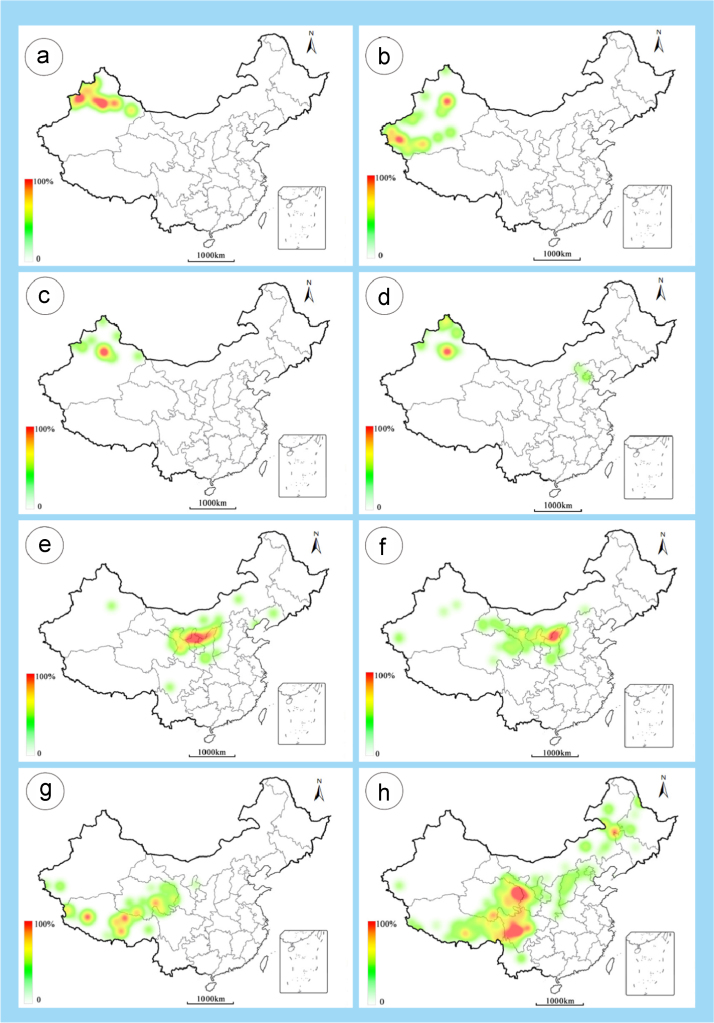
a. *Seriphidium borotalense*; b. *Seriphidium rhodanthum*; c. *Seriphidium kaschgaricum*; d. *Seriphidium terrae-albae*; e. *Artemisia ordosica*; f. *Artemisia sphaerocephala*; g. *Artemisia nanschanica*; h. *Artemisia desertorum*. The colour of the bars from red to white representing the probability of the species occurrence decreasing.

**Fig. 4:**
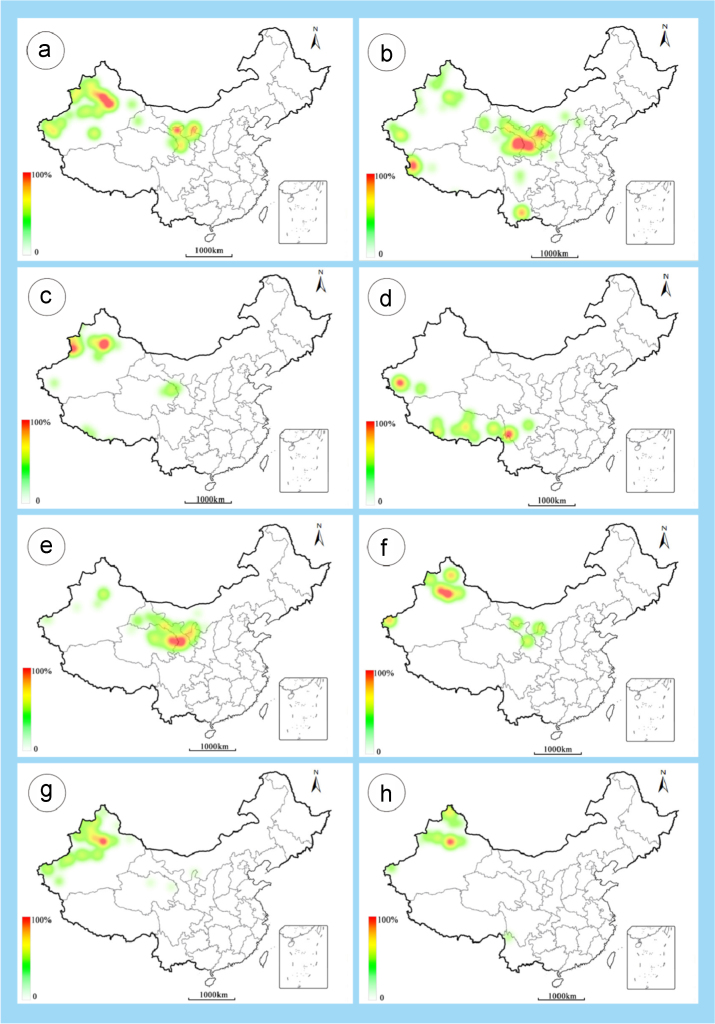
a. *Karelinia caspia*; b. *Ajania fruticulosa*; c. *Ajania fastigiata*; d. *Ajania tibetica*; e. *Asterothamnus centrali-asiaticus*; f. *Cistanche deserticola*; g. *Anabasis aphylla*; h. *Anabasis salsa*. The colour of the bars from red to white representing the probability of the species occurrence decreasing.

**Fig. 5:**
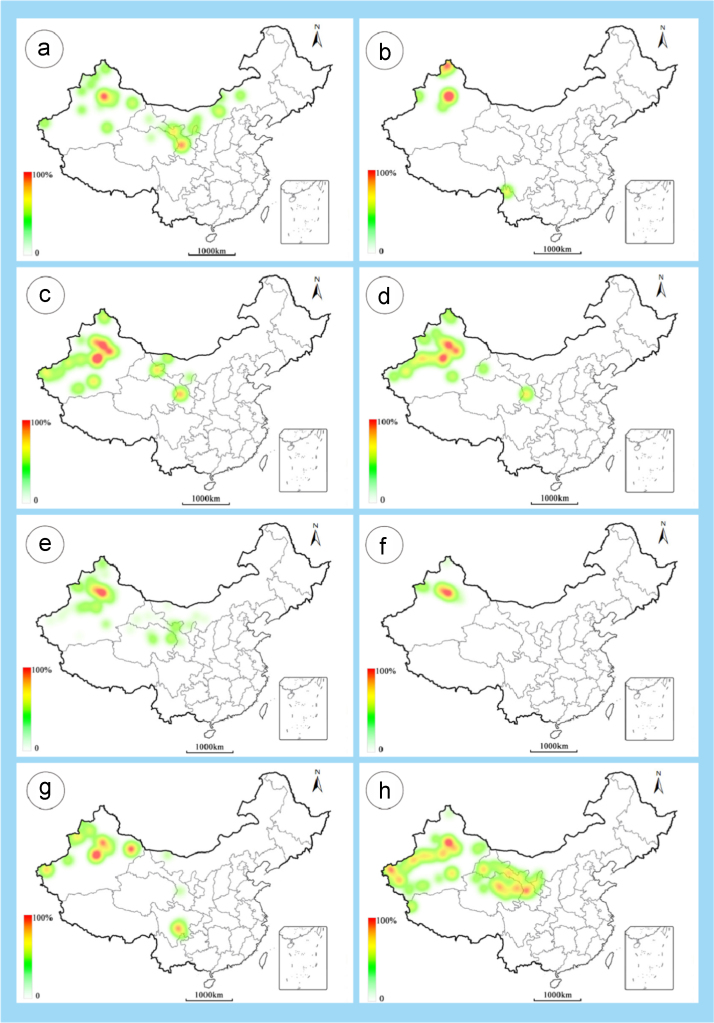
a. *Anabasis brevifolia*; b. *Atriplex cana*; c. *Halostachys caspica*; d. *Halocnemum strobilaceum*; e. *Haloxylon ammodendron*; f. *Haloxylon persicum*; g. *Iljinia regelii*; h. *Sympegma regelii*. The colour of the bars from red to white representing the probability of the species occurrence decreasing.

**Fig. 6:**
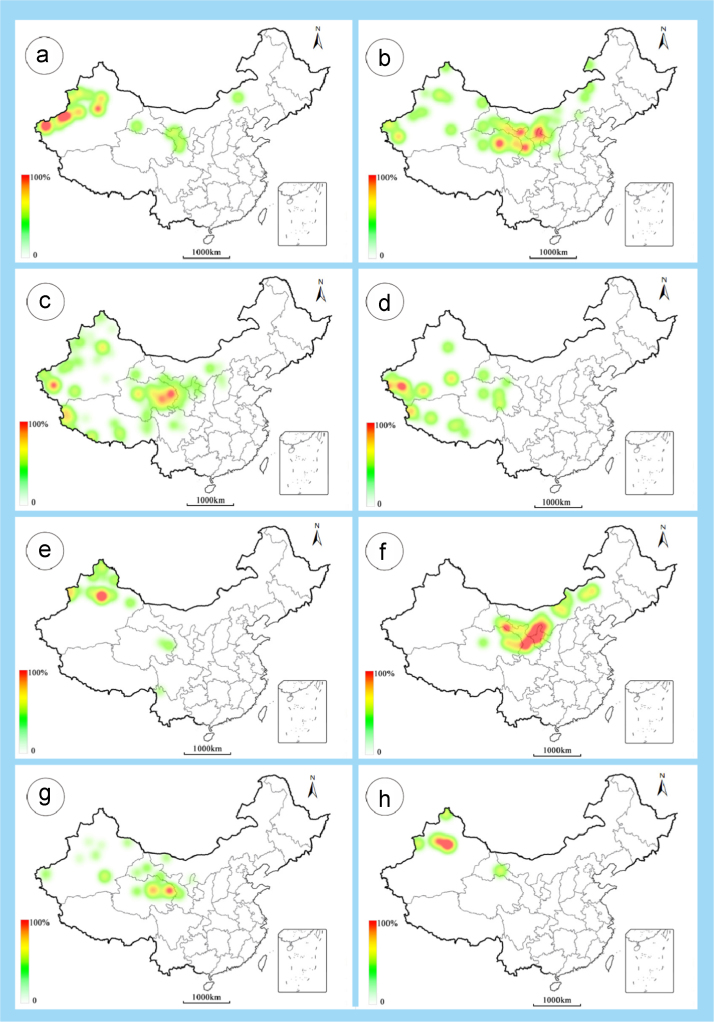
a. *Kalidium schrenkianum*; b. *Kalidium cuspidatum*; c. *Krascheninnikovia compacta*; d. *Krascheninnikovia ceratoides*; e. *Nanophyton erinaceum*; f. *Salsola passerina*; g. *Salsola abrotanoides*; h. *Suaeda physophora*. The colour of the bars from red to white representing the probability of the species occurrence decreasing.

**Fig. 7:**
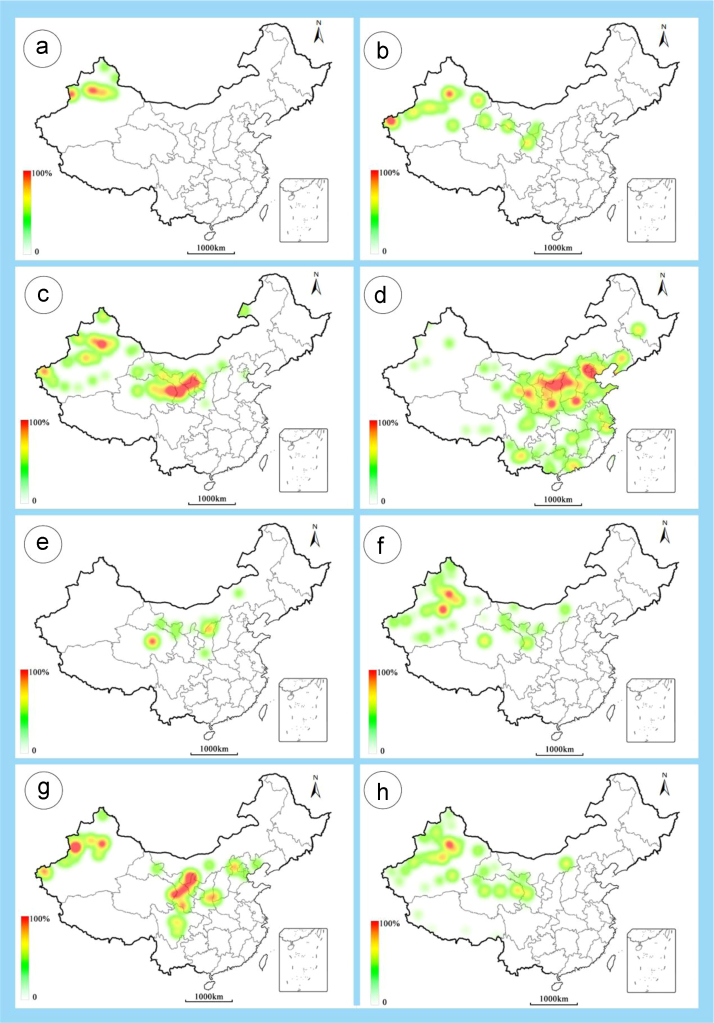
a. *Suaeda microphylla*; b. *Gymnocarpos przewalskii*; c. *Reaumuria soongarica*; d. *Tamarix chinensis*; e. *Psammochloa villosa*; f. *Populus euphratica*; g. *Convolvulus tragacanthoides*; h. *Ephedra przewalskii*. The colour of the bars from red to white representing the probability of the species occurrence decreasing.

See Fig. 1, Fig. 2, Fig. 3, Fig. 4, Fig. 5, Fig. 6, Fig. 7.

## 2 Experimental design, materials, and methods

### 2.1 Experimental design

Here, we attempt to 1) depict a pollen spectrum of the dominant species in desert vegetation to improve the resolution and accuracy of pollen identification in the eastern ACA, and 2) plot distribution maps of each dominant species for a better understanding of the distribution patterns of these desert plants [1].

### 2.2 Materials

Pollen grains of 56 species were extracted from voucher specimens (Table 2) from the PE herbarium at the Institute of Botany (herbarium code: PE). The distribution maps are plotted using the Google Maps and the species distribution data at the county level supplied by the Chinese Virtual Herbarium (http://www.cvh.ac.cn/).

**Table 2:** List of the voucher specimen in PE Herbarium, Institute of Botany, Chinese Academy of Sciences.

**No.**	**Family**	**Genus**	**Species**	**Coll. No.**	**Specimen No.**	**Distribution**
1	Asteraceae	*Ajania*	*A. fruticulosa*	7859	00386862	Fig. 4b
2			*A. fastigiata*	522	00386826	Fig. 4c
3			*A. tibetica*	12808	00420246	Fig. 4d
4		*Artemisia*	*A. ordosica*	H.3194	00445889	Fig. 3e
5			*A. sphaerocephala*	44	01894259	Fig. 3f
6			*A. nanschanica*	W.5650	00445844	Fig. 3g
7			*A. desertorum*	6339	00421401	Fig. 3h
8		*Asterothamnus*	*A. centrali-asiaticus*	L.2318	00301799	Fig. 4e
9		*Karelinia*	*K. caspia*	3696	01577791	Fig. 4a
10		*Seriphidium*	*S. santolinum*	3797	00457656	Fig. 2h
11			*S. borotalense*	5141	00457452	Fig. 3a
12			*S. rhodanthum*	116	00457852	Fig. 3b
13			*S. kaschgaricum*	74-1434	00457555	Fig. 3c
14			*S. terrae-albae*	2977	00457814	Fig. 3d
15	Caryophyllaceae	*Gymnocarpos*	*G. przewalskii*	Tazhong0010	00583459	Fig. 7b
16	Chenopodiaceae	*Anabasis*	*A. aphylla*	h29	00120575	Fig. 4g
17			*A. brevifolia*	80-459	00120685	Fig. 5a
18			*A. salsa*	1-363	00147264	Fig. 4h
19		*Atriplex*	*A. cana*	6978	00120126	Fig. 5b
20		*Halocnemum*	*H. strobilaceum*	15	00540950	Fig. 5d
21		*Halostachys*	*H. caspica*	253	00146286	Fig. 5c
22		*Haloxylon*	*H. ammodendron*	148	00541353	Fig. 5e
23			*H. persicum*	s.n.	00541406	Fig. 5f
24		*Iljinia*	*I. regelii*	502	01196845	Fig. 5g
25		*Kalidium*	*K. cuspidatum*	8743	00541652	Fig. 6b
26			*K. schrenkianum*	9778	00509851	Fig. 6a
27		*Krascheninnikovia*	*K. compacta*	609	00540518	Fig. 6c
28			*K. latens*	13666	00147145	Fig. 6d
29		*Nanophyton*	*N. erinaceum*	6673	00526712	Fig. 6e
30		*Salsola*	*S. abrotanoides*	11593	00145945	Fig. 6g
31			*S. passerina*	200	00528119	Fig. 6f
32		*Suaeda*	*S. microphylla*	3408	00527362	Fig. 7a
33			*S. physophora*	4534	00527387	Fig. 6h
34		*Sympegma*	*S. regelii*	305	00527736	Fig. 5h
35	Cistaceae	*Helianthemum*	*H. songaricum*	151	01176401	Fig. 2g
36	Convolvulaceae	*Convolvulus*	*C. tragacanthoides*	2689	00112946	Fig. 7g
37	Ephedraceae	*Ephedra*	*E. przewalskii*	11605	01606079	Fig. 7h
38	Fabaceae	*Alhagi*	*A. sparsifolia*	11613	01468293	Fig. 2f
39		*Ammodendron*	*A. bifolium*	SW7400216	00099928	Fig. 2d
40		*Ammopiptanthus*	*A. mongolicus*	1537	00099950	Fig. 2c
41		*Caragana*	*C. korshinskii*	10029	00180727	Fig. 2e
42	Nitrariaceae	*Nitraria*	*N. roborowskii*	123	00965946	Fig. 1b
43			*N. sphaerocarpa*	101	00973240	Fig. 1c
44		*Peganum*	*P. harmala*	86013	00972516	Fig. 1a
45	Orobanchaceae	*Cistanche*	*C. deserticola*	6499	01542440	Fig. 4f
46	Poaceae	*Psammochloa*	*P. villosa*	89	00614742	Fig. 7e
47	Polygonaceae	*Calligonum*	*C. leucocladum*	F208	01050284	Fig. 1h
48			*C. mongolicum*	1005	00041439	Fig. 1g
49			*C. roborowskii*	55	00041496	Fig. 1f
50			*C. rubicundum*	74-1092	00041901	Fig. 2a
51	Rosaceae	*Potaninia*	*P. mongolica*	10271	00604890	Fig. 2b
52	Salicaceae	*Populus*	*P. euphratica*	64-001	00562476	Fig. 7f
53	Tamaricaceae	*Reaumuria*	*R. soongarica*	133	01177164	Fig. 7c
54		*Tamarix*	*T. chinensis*	s.n.	01177582	Fig. 7d
55	Zygophyllaceae	*Tetraena*	*T. mongolica*	s.n.	00972777	Fig. 1d
56		*Zygophyllum*	*Z. xanthoxylon*	2108	00973420	Fig. 1e

### 2.3 Methods and terminology

Pollen grains were acetolyzed by the standard method [2] and fixed in glycerin jelly. Standard procedures were followed for light microscopy (LM) and scanning electron microscopy (SEM). All pollen grains were observed and photographed at a magnification of ×400 or 1000 under a Leica 4000 instrument. At least 20 pollen grains were measured for each species. The average values and the variation ranges were used to describe the pollen morphological characters. The fine characteristics of exine ornamentation were observed under SEM.

The pollen morphological terminology follows the overview of Hesse et al. [3] and Punt et al. [4]. For instance, Punt et al. [4] divided the pollen shapes into prolate (1.33–2.00), subprolate (1.14–1.33), spheroidal (0.88–1.14), and suboblate (0.75–0.88) based on their *P*/*E* ratio values. The *P*/*E* ratio of each pollen grain was calculated using the polar axis diameter (*P*) and equatorial diameter (*E*). Hesse et al. [3] defined pollen size as very small (<10 μm), small (10–25 μm), medium (26–50 μm), large (51–100 μm), and very large (> 100 μm) based on the pollen diameters.
