# Supplementary material for: Dataset of pollen morphological traits of 56 dominant species among desert vegetation in the eastern arid central Asia
Source: Data Brief. 2018 Mar 31;18:1022–46. doi: 10.1016/j.dib.2018.03.122 (PMC5996618; doi:10.1016/j.dib.2018.03.122)
Supplement: Supplementary file 2 — Supplementary material [file mmc2.doc]

The detailed descriptions of the pollen morphology of the 56 species (Plates 1-11) are listed as below.

**Nitrariaceae**

*Peganum harmala* L. (Plate 1a-c)

Pollen grains spheroidal, 23.5 (21.5-25.1) μm × 21.5 (19-23.3) μm, small size. P/E = 1.09 (1.05-1.16). Elliptical in equatorial view and trilobate circular in polar view. Apertures tricolporate. Colpi long, nearly reaching the poles, sunken, broad, membrane granulate, margin regular. Exine ca. 1.7 (1.5-2.0) μm. Sexine as thick as nexine. Ornamentation reticulate. Mesh and muri obvious. Lumina heterobrochate.

Distribution: Ningxia, Inner Mongolia, Tibet, Xinjiang, Hexi in Gansu Province (Fig. 1a), Mongolia, central Asia, western Asia, Iran to India (northwest), the Mediterranean, and North Africa. Habitats: Arid grassland in desert regions, oasis edge low salinization, sand hill or river sand dunes (up to 3600 m).

*Nitraria roborowskii* Kom. (Plate 1d-f)

Pollen grains prolate, 34.5 (31.4-36.8) μm × 22.0 (19.6-24.2) μm, medium size. P/E = 1.57 (1.42-1.83). Elliptical in equatorial view and trilobate circular in polar view. Apertures tricolporate. Pore diamond-shaped, colpi long, nearly reaching the poles, sunken, membrane granulate, margin regular. Exine ca. 2.7 (2.1-3.3) μm. Exine thick at the poles. Sexine thicker than nexine. Ornamentation striate.

Distribution: Desert areas in the west of Inner Mongolia, Ningxia, Hexi in Gansu, Qinghai and Xinjiang (Fig. 1b), and Mongolia. Habitats: Lake basin margins and sand of oasis periphery.

*Nitraria sphaerocarpa* Maxim. (Plate 1g-i)

Pollen grains prolate, 33.9 (28.8-38.5) μm × 23.8 (21.8-26.7) μm, medium size. P/E = 1.42 (1.29-1.7). Elliptical in equatorial view and trilobate circular in polar view. Apertures tricolporate. Pore diamond-shaped, colpi long, nearly reaching the poles, sunken, membrane granulate, margin regular. Exine ca. 2.5 (2.1-2.9) μm. Exine thick at the poles. Sexine thicker than nexine. Ornamentation striate.

Distribution: Western Inner Mongolia, Xinjiang, Hexi in Gansu (Fig. 1c), and Mongolia. Habitats: Gobi piedmont plains and flat gravel sand.

**Zygophyllaceae**

*Tetraena mongolica* Maxim. (Plate 1j-l)

Pollen grains subprolate, 15.0 (13.1-16.3) μm × 12.6 (11.5-13.7) μm, small size. P/E = 1.19 (1.09-1.33). Elliptical in equatorial view and trilobate circular in polar view. Apertures tricolporate. Colpi long, nearly reaching the poles, sunken, narrow, margin regular. Exine ca. 1.1 (0.8-1.4) μm. Ornamentation reticulate (SEM). Mesh and muri obvious. Lumina heterobrochate.

Distribution: Inner Mongolia (Fig. 1d). Habitats: Steppe-desert of the Yellow River terraces and low mountain slopes.

*Zygophyllum xanthoxylon (Bunge) Maxim.* (Plate 1m-o)

Pollen grains subprolate, 19.0 (16.3-23.2) μm × 16.5 (13.8-20.8) μm, small size. P/E = 1.16 (1.03-1.38). Elliptical in equatorial view and trilobate circular in polar view. Apertures tricolporate. Colpi long, nearly reaching the poles, sunken, broad, margin regular. Exine ca. 1.6 (1.3-2.1) μm thick. Ornamentation reticulate. Mesh and muri obvious. Lumina smaller near the colpi.

Distribution: Western Inner Mongolia, western Gansu, western Ningxia, Xinjiang, Qinghai (Fig. 1e), and Mongolia. Habitats: Desert and semi-desert gravel river terraces, low mountain slopes, gravel hills and piedmont plains.

**Polygonaceae**

*Calligonum* *roborowskii* Losinsk. (Plate 2a-c)

Pollen grains spheroidal, 32.3 (30.1-37.8) μm × 28.3 (25.5-32.8) μm, medium size. P/E = 1.05 (1.01-1.19). Elliptical in equatorial view and trilobate circular in polar view. Apertures tricolporate. Colpi long, reaching the poles, sunken, margin regular. Exine ca. 2.6 (2.3-3.1) μm. Circular endoapertures obvious (LM). Ornamentation reticulate-foveolate with circular lumina (SEM).

Distribution: Western Gansu, southern and eastern Xinjiang (Fig. 1f). Habitats: Diluvial fans, gritty desert, sand of gravel desert, alluvial plains and dry valleys (above 900-1500 m).

*Calligonum mongolicum* Turcz. (Plate 2d-f)

Pollen grains spheroidal, 35.9 (34.5-38.6) μm × 34.1 (32.4-35.4) μm, medium size. P/E = 1.09 (0.96-1.27). Elliptical in equatorial view and trilobate circular in polar view. Apertures tricolporate. Colpi long, reaching the poles, sunken, margin regular. Exine ca. 2.3 (1.9-2.9) μm. Circular apertures obvious (LM). Ornamentation reticulate-foveolate, with circular lumina (SEM).

Distribution: Central and western Inner Mongolia, western Gansu and eastern Xinjiang (Fig. 1g), and Mongolia. Habitats: Active, semi-fixed and fixed dunes, sand, gritty desert, accumulations of coarse sand (above 500-1800 m).

*Calligonum leucocladum* (Schrenk) Bunge (Plate 2g-i)

Pollen grains spheroidal, 30.4 (26.7-32.1) μm × 27.4 (22.8-30.5) μm, medium size. P/E = 1.11 (1.03-1.17). Elliptical in equatorial view and trilobate circular in polar view. Apertures tricolporate. Colpi long, reaching the poles, sunken, margin regular. Exine ca. 2.4 (1.6-2.8) μm. Circular apertures obvious (LM). Ornamentation reticulate-foveolate, with circular lumina (SEM).

Distribution: Northern Tianshan Mountains in Xinjiang (Fig. 1h), and Kazakhstan.

Habitats: Semi-fixed and fixed sand dunes (500-1200 m).

*Calligonum rubicundum* Bunge (Plate 2j-l)

Pollen grains spheroidal, 33.5 (26.9-40.2) μm × 29.7 (23.9-35.9) μm, medium size. P/E ratio 1.13 (1.04-1.43). Elliptical in equatorial view and trilobate circular in polar view. Apertures tricolporate. Colpi long, reaching the poles, sunken, margin regular. Exine ca. 2.3 (1.8-2.9) μm thick. Ornamentation psilate (LM), foveolate (SEM). Circular apertures obvious (LM). Ornamentation reticulate-foveolate, with circular lumina (SEM).

Distribution: Northwest Xinjiang (Fig. 2a), Russia, and Kazakhstan. Habitats: Semi-fixed and fixed sand dunes (450-1000 m).

**Rosaceae**

*Potaninia mongolica* Maxim. (Plate 2m-o)

Pollen grains subprolate, 20.3 (16.3-25.4) μm × 17.9 (13.3-20.8) μm, small size. P/E = 1.14 (1.02-1.35). Elliptical in equatorial view and trilobate circular in polar view. Apertures tricolporate. Colpi long, nearly reaching the poles, sunken. Exine ca. 1.7 (1.2-2.2) μm. Ornamentation striate (SEM).

Distribution: Inner Mongolia of China (Fig. 2b) and Mongolia. Habitats: Sandy desert.

**Fabaceae**

*Ammopiptanthus mongolicus* (Kom.) S. H. Cheng (Plate 3a-c)

Pollen grains subprolate, 21.1 (15.3-23.3) μm × 16.9 (13.3-18.4) μm, small size. P/E ratio 1.25 (1.15-1.37). Elliptical in equatorial view and circular in polar view. Apertures tricolporate. Colpi long, reaching the poles, broad, margin irregular. Exine ca. 1.3 (1.1-1.4) μm thick. Ornamentation psilate (LM), reticulate (SEM). Mesh small. Apertures obvious.

Distribution: Inner Mongolia, Ningxia, Gansu and Southern Mongolia (Fig. 2c). Habitats: Dunes, platform adjacent to [riverbank](http://dict.youdao.com/w/river bank/" \l "keyfrom=E2Ctranslation).

*Ammodendron bifolium* (Pall.) Yakovlev (Plate 3d-f)

Pollen grains spheroidal, 17.9 (15.9-20.4) μm × 17.1 (14.9-18.9) μm, small size. P/E ratio 1.05 (0.91-1.14). Circular in both equatorial and polar view. Apertures tricolporate. Colpi long, reaching the poles, broad, margin irregular. Exine ca. 1.4 (1.1-1.7) μm thick. Ornamentation psilate (LM), reticulate (SEM). Mesh small. Apertures obvious.

Distribution: Xinjiang (Fig. 2d). Habitats: Dry gritty sand belts.

*Caragana korshinskii* Kom. (Plate 3g-i)

Pollen grains subprolate, 21.3 (17.8-25.5) μm × 17.4 (13.3-21.9) μm, small size. P/E ratio 1.24 (1.01-1.82). Elliptical in equatorial view and circular in polar view. Apertures tricolporate. Colpi long, reaching the poles, broad, margin regular. Exine ca. 1.6 (1.2-2.1) μm thick. Ornamentation psilate (LM), reticulate (SEM). Mesh small.

Distribution: Inner Mongolia, Ningxia and Gansu (Fig. 2e). Habitats: Semi-fixed and fixed sand bodies.

*Alhagi sparsifolia* Shap. (Plate 3j-l)

Pollen grains spheroidal, 13.7 (13.1-17.1) μm × 13.7 (11.9-15.3) μm, small size. P/E ratio 1.08 (0.96-1.37). Circular in both equatorial and polar view. Apertures tricolporate. Colpi long, reaching the poles, broad, margin regular. Exine ca. 1.2 (1-1.5) μm thick. Ornamentation psilate (LM), reticulate (SEM). Mesh small.

Distribution: Inner Mongolia, Qinghai, Gansu, Xinjiang (Fig. 2f), Kazakhstan, Uzbekistan, Turkmenistan, Kyrgyzstan, and Tajikistan.

Habitats: Sand of desert region, bank and edge of farmland.

**Cistaceae**

*Helianthemum songaricum* Schrenk ex Fisch. & C. A. Mey. (Plate 3m-o)

Pollen grains spheroidal, 39.6 (37.8-40.4) μm × 37.6 (33.7-39.8) μm, medium size. P/E ratio 1.05 (1.01-1.12). Circular in both equatorial and polar view. Apertures tricolporate. Colpi long, reaching the poles, margin regular. Exine ca. 3.9 (3.2-4.8) μm thick. Ornamentation reticulate (LM and SEM).

Distribution: Inner Mongolia, Gansu, Xinjiang (Fig. 2g), and [Central Asia](http://dict.youdao.com/w/Central Asia/" \l "keyfrom=E2Ctranslation).

Habitats: [Steppe](http://dict.youdao.com/w/steppe/#keyfrom=E2Ctranslation)-desert, rocky and gravelly slopes.

**Asteraceae**

*Seriphidium santolinum* (Schrenk) Poljakov (Plate 4a-c)

Pollen grains spheroidal, 22.7 (20.5-25.1) μm × 23.1 (20.9-26.1) μm, small size. P/E ratio 0.98 (0.87-1.07). Circular in equatorial view and trilobate circular in polar view. Apertures tricolporate. Colpi long, reaching the poles, margin regular. Exine ca. 3.0 (2.6-3.5) μm thick. Ornamentation psilate (LM), microechinate (SEM).

Distribution: Northern Xinjiang (Fig. 2h) and the desert area in [Central Asia](http://dict.youdao.com/w/Central Asia/" \l "keyfrom=E2Ctranslation), Iran and Caucasus. Habitats: Semi-fixed and fixed sands (above 1400 m).

*Seriphidium borotalense* (Poljakov) Ling & Y. R. Ling (Plate 4d-f)

Pollen grains spheroidal, 19.6 (17.4-22.7) μm × 19.4 (14.6-21.2) μm, small size. P/E ratio 1.02 (0.91-1.26). Circular in equatorial view and trilobate circular in polar view. Apertures tricolporate. Colpi long, reaching the poles, margin regular. Exine ca. 2.8 (1.7-3.5) μm thick. Ornamentation psilate (LM), microechinate (SEM).

Distribution: Northern Xinjiang (Fig. 3a). Habitats: Desert or semi-desert grasslands, Gobi, gravelly slopes and diluvial fans (1000-1500 m).

*Seriphidium rhodanthum* (Rupr.) Poljakov (Plate 4g-i)

Pollen grains spheroidal, 22.5 (19.6-29.5) μm × 22 (20.4-24.2) μm, small size. P/E ratio 1.02 (0.90-1.22). Circular in equatorial view and trilobate circular in polar view. Apertures tricolporate. Colpi long, reaching the poles, margin regular. Exine ca. 2.6 (2-3.8) μm thick. Ornamentation psilate (LM), microechinate (SEM).

Distribution: Southwest Xinjiang (Fig. 3b) and Eastern Central Asia. Habitats: Gravelly slopes, steppes and terraces (1500-3700 m).

*Seriphidium kaschgaricum* (Krasch.) Poljakov (Plate 4j-l)

Pollen grains spheroidal, 21.9 (20.4-24.5) μm × 21.3 (19.5-22.6) μm, small size. P/E ratio 1.03 (0.95-1.21). Circular in equatorial view and trilobate circular in polar view. Apertures tricolporate. Colpi long, reaching the poles, margin regular. Exine ca. 3.1 (2.5-3.7) μm thick. Ornamentation psilate (LM), microechinate (SEM).

Distribution: Northern Xinjiang (Fig. 3c) and Eastern Central Asia. Habitats: Gravelly slopes, Gobi, dry river valleys, and river gravel beaches and roadsides (below 1200 m).

*Seriphidium terrae-albae* (Krasch.) Poljakov (Plate 4m-o)

Pollen grains spheroidal, 21.4 (18.0-26.1) μm × 21.1 (17.7-27.0) μm, small size. P/E ratio 1.01 (0.93-1.12). Circular in equatorial view and trilobate circular in polar view. Apertures tricolporate. Colpi long, reaching the poles, margin regular. Exine ca. 2.8 (1.9-3.8) μm thick. Ornamentation psilate (LM), microechinate (SEM).

Distribution: Northern Xinjiang, Mongolia (Fig. 3d) and Central Asia. Habitats: Desert and desert edges, gravelly Gobi areas.

*Artemisia ordosica* Krasch. (Plate 5a-c)

Pollen grains spheroidal, 20.5 (17.6-23.5) μm × 18.3 (17.0-21.3) μm, small size. P/E ratio 1.12 (0.95-1.28). Circular in equatorial view and trilobate circular in polar view. Apertures tricolporate. Colpi long, reaching the poles, margin regular. Exine ca. 2.4 (1.9-2.8) μm thick. Ornamentation psilate (LM), microechinate (SEM).

Distribution: North and west of China (Fig. 3e). Habitats: Semi-fixed or fixed sand dunes, steppe and dry slopes, as well as desert and semi-desert areas (below 1500 m).

*Artemisia sphaerocephala* Krasch. (Plate 5d-f)

Pollen grains spheroidal, 23.2 (17.6-25.3) μm × 21.0 (16.0-23.9) μm, small size. P/E ratio 1.11 (1.00-1.29). Circular in equatorial view and trilobate circular in polar view. Apertures tricolporate. Colpi long, reaching the poles, margin regular. Exine ca. 3.0 (2.3-3.6) μm thick. Ornamentation psilate (LM), microechinate (SEM).

Distribution: Inner Mongolia (southwest), Shanxi (north), northern Shaanxi, Ningxia and Gansu (north, west), Qinghai (north), Xinjiang (east) (Fig. 3f), and Mongolia. Habitats: Desert region of active and semi-fixed or fixed sand dunes and dry slopes (1000-2850 m).

*Artemisia nanschanica* Krasch. (Plate 5g-i)

Pollen grains spheroidal, 20.2 (17.5-24.4) μm × 18.1 (15.3-20.1) μm, small size. P/E ratio 1.12 (0.92-1.35). Circular in equatorial view and trilobate circular in polar view. Apertures tricolporate. Colpi long, reaching the poles, margin regular. Exine ca. 2.5 (1.9-2.9) μm thick. Ornamentation psilate (LM), microechinate (SEM).

Distribution: Qinghai, Gansu (south), Xinjiang (south) and Tibet (Fig. 3g). Habitats: Beaches, dry mountain slopes, grassland, sandy and gravelly slopes (2100-5300 m).

*Artemisia desertorum* Spreng. (Plate 5j-l)

Pollen grains spheroidal, 20.9 (16.7-24.7) μm × 19.0 (14.6-21.0) μm, small size. P/E ratio 1.10 (0.95-1.36). Circular in equatorial view and trilobate circular in polar view. Apertures tricolporate. Colpi long, reaching the poles, margin regular. Exine ca. 2.7 (2.2-3.0) μm thick. Ornamentation psilate (LM), microechinate (SEM).

Distribution: China (Fig. 3h), Korea, Japan, India (north), Pakistan (north) and Central Asia (east). Habitats: Grasslands, meadow and forest grasslands, montane grassland, gravelly slopes, dry river valleys, river beaches, forest margins, and roadsides.

*Karelinia caspia* (Pall.) Less. (Plate 5m-o)

Pollen grains spheroidal, 25.0 (18.7-29.6) μm × 23.7 (19.8-26.7) μm, medium size. P/E ratio 1.07 (0.88-1.23). Apertures tricolporate. Colpi long, reaching the poles, endoapertures obvious. Exine ca. 3.3 (2.6-4.1) μm thick. Spine length 4.8 (3.8-5.7) μm, base diameter 3.5 (3.1-4.3) μm. Ornamentation echinate (LM), echinate-perforate (SEM).

Distribution: West and north of China (Fig. 4a), Mongolia, Central Asia, Eastern Europe, Iran and Turkey. Habitats: Gobi beaches, sand dunes, saline meadows and reed-adjacent paddy fields.

*Ajania fruticulosa* (Ledeb.) Poljakov (Plate 6a-c)

Pollen grains spheroidal, 24.4 (22.7-26.4) μm × 23.3 (21.2-26.2) μm, small size. P/E ratio 1.05 (0.96-1.14). Apertures tricolporate. Colpi long, reaching the poles, endoapertures obvious. Exine ca. 3.6 (2.6-5.0) μm thick. Spine length 2.4 (1.9-2.7) μm, base diameter 2.9 (2.1-3.5) μm. Ornamentation echinate (LM), echinate-perforate (SEM).

Distribution: West and north of China (Fig. 4b) and Central Asia. Habitats: Desert and desert steppe (550-4400 m).

*Ajania fastigiata* (C. Winkl.) Poljakov (Plate 6d-f)

Pollen grains spheroidal, 27.0 (22.2-31.9) μm × 25.3 (18.6-31.9) μm, medium size. P/E ratio 1.07 (0.97-1.26). Apertures tricolporate. Colpi long, reaching the poles, endoapertures obvious. Exine ca. 3.8 (2.8-5.5) μm thick. Spine length 2.8 (2.1-3.2) μm, base diameter 3.0 (2.7-3.5) μm. Ornamentation echinate (LM), echinate-perforate (SEM).

Distribution: Xinjiang (Fig. 4c), Siberia, Central Asia, and Mongolia. Habitats: Steppe, semi-desert and forest (900-2260 m).

*Ajania tibetica* (Hook.f. & Thomson) Tzvelev (Plate 6g-i)

Pollen grains spheroidal, 23.2 (21.0-26.7) μm × 22.2 (20.3-26.0) μm, small size. P/E ratio 1.05 (0.91-1.17). Apertures tricolporate. Colpi long, reaching the poles, endoapertures obvious. Exine ca. 3.3 (2.7-4.7) μm thick. Spine length 1.0 (0.7-1.1) μm, base diameter 0.9 (0.7-1.1) μm. Ornamentation echinate (LM), echinate-perforate (SEM).

Distribution: South and west of China (Fig. 4d), northern India and Central Asian region. Habitats: Mountain slopes (3900-4700 m).

*Asterothamnus centrali-asiaticus* Novopokr. (Plate 6j-l)

Pollen grains spheroidal, 28.2 (25.5-36.0) μm × 26.9 (24.0-31.3) μm, medium size. P/E ratio 1.05 (0.86-1.18). Apertures tricolporate. Colpi long, reaching the poles, endoapertures obvious. Exine ca. 2.6 (2.2-3.1) μm thick. Spine length 2.5 (1.9-2.9) μm, base diameter 2.3 (1.9-2.6) μm. Ornamentation echinate (LM), echinate-perforate (SEM).

Distribution: West and south of China (Fig. 4e) and southern Mongolia. Habitats: Grasslands or desert areas.

**Orobanchaceae**

*Cistanche deserticola* Y. C. Ma (Plate 6m-o)

Pollen grains subprolate, 28.5 (24.7-34.1) μm × 23.9 (16.3-28.1) μm, medium size. P/E ratio 1.21 (0.91-1.45). Elliptical in equatorial view and trilobate circular in polar view. Apertures tricolpate. Colpi long, nearly reaching the poles, wide, margin regular. Exine ca. 1.8 (1.3-2.5) μm thick. Ornamentation psilate (LM), reticulate (SEM). Lumina heterobrochate.

Distribution: Northwestern China (Fig. 4f). Habitat: Sand dunes in Sacsaoul desert (225-1150 m).

**Chenopodiaceae**

*Anabasis aphylla* L. (Plate 7a-c)

Pollen grains spheroidal, 17.7 (14.9-20.9) μm × 16.9 (14.6-20.2) μm, small size. P/E ratio 1.05 (1.01-1.12). Apertures pantoporate with 18 (14-20) pores, pores circular approximately 2.7 (2.3-3.3) μm in diameter evenly distributed on the surface of grains, echinate with granulae. Exine ca. 2.0 (1.4-2.5) μm thick. Ornamentation granulate (LM), microechinate-perforate (SEM).

Distribution: Northwest China (Fig. 4g), Europe, Central Asia, and Siberia. Habitats: Gobi, piedmont alluvial fans, gravel dunes and arid hillsides.

*Anabasis salsa* (Ledeb.) Benth. ex Volkens (Plate 7d-f)

Pollen grains spheroidal, 18.4 (13.8-24.8) μm × 17.9 (13.3-22.9) μm, small size. P/E ratio 1.03 (1.01-1.09). Apertures pantoporate with 16 (12-22) pores, pores circular approximately 3.4 (2.9-3.8) μm in diameter evenly distributed on the surface of grains, echinate with granules. Exine ca. 1.9 (1.6-2.3) μm thick. Ornamentation granulate (LM), microechinate-perforate (SEM).

Distribution: Northwest China, Mongolia (Fig. 4h), Caucasus, Kazakhstan, and Siberia. Habitats: Gobi and saline deserts.

*Anabasis brevifolia* C. A. Mey. (Plate 7g-i)

Pollen grains spheroidal, 19.2 (16.2-20.9) μm × 18.3 (14.9-20.7) μm, small size. P/E ratio 1.05 (1.01-1.09). Apertures pantoporate with 18 (16-20) pores, pores circular approximately 2.5 (2-3.6) μm in diameter evenly distributed on the surface of grains, echinate with granules. Exine ca. 2 (1.7-2.3) μm thick. Ornamentation granulate (LM), microechinate-perforate (SEM).

Distribution: Northwest China (Fig. 5a), Mongolia, Siberia, and Kazakhstan. Habitats: Gobi, alluvial fans and arid hillsides.

*Atriplex cana* Ledeb. (Plate 7j-l)

Pollen grains spheroidal, 19.2 (16.3-22.2) μm × 18.6 (14.8-21.2) μm, small size. P/E ratio 1.04 (1-1.1). Apertures pantoporate with 32 (24-36) pores, pores circular approximately 2.1 (1.6-2.7) μm in diameter evenly distributed on the surface of grains, echinate with granules. Exine ca. 2.1 (1.8-2.5) μm thick. Ornamentation granulate (LM), microechinate-perforate (SEM).

Distribution: Northwest China (Fig. 5b), Kazakhstan and Siberia. Habitats: Arid hillsides, semi-desert and lakesides.

*Halostachys caspica* C. A. Mey. (Plate 7m-o)

Pollen grains spheroidal, 18.0 (14.7-20.9) μm × 16.9 (14.5-20.6) μm, small size. P/E ratio 1.06 (1.01-1.15). Apertures pantoporate with 28 (24-34) pores, pores circular approximately 1.8 (1.4-2) μm in diameter evenly distributed on the surface of grains, echinate with granules. Exine ca. 1.8 (1.4-2.3) μm thick. Ornamentation granulate (LM), microechinate-perforate (SEM).

Distribution: Northwest China (Fig. 5c), Iran, Afghanistan, and Mongolia. Habitats: Saline-alkali beaches, river valleys and saline lakesides.

*Halocnemum strobilaceum* (Pall.) M. Bieb. (Plate 8a-c)

Pollen grains spheroidal, 17.3 (14.8-18.8) μm × 16.5 (13.5-18.4) μm, small size. P/E ratio 1.05 (1.01-1.13). Apertures pantoporate with 26 (20-30) pores, pores circular approximately 1.7 (1-2.3) μm in diameter evenly distributed on the surface of grains, echinate with granules. Exine ca. 1.9 (1.5-2.3) μm thick. Ornamentation granulate (LM), microechinate-perforate (SEM).

Distribution: Northwest China (Fig. 5d), Central Asia, Mongolia, Afghanistan, Iran, and northern Africa. Habitats: Saline beaches and saline wetlands.

*Haloxylon ammodendron* (C. A. Mey.) Bunge ex Fenzl (Plate 8d-f)

Pollen grains spheroidal, 19.2 (15.9-21.6) μm × 18.8 (15.3-21.4) μm, small size. P/E ratio 1.02 (1-1.05). Apertures pantoporate with 22 (18-24) pores, pores circular approximately 2.8 (2.5-3.1) μm in diameter evenly distributed on the surface of grains, echinate with granules. Exine ca. 1.4 (1.2-1.9) μm thick. Ornamentation granulate (LM), microechinate-perforate (SEM).

Distribution: Northwest China (Fig. 5e), Central Asia and Siberia. Habitats: Sand dunes, saline-alkali deserts, river sands.

*Haloxylon persicum* Bunge (Plate 8g-i)

Pollen grains spheroidal, 19.2 (11.7-19.3) μm × 16.1 (11.4-18.9) μm, small size. P/E ratio 1.03 (1.01-1.12). Apertures pantoporate with 20 (12-24) pores, pores circular approximately 2.3 (1.8-2.8) μm in diameter evenly distributed on the surface of grains, echinate with granules. Exine ca. 1.6 (1.2-2.0) μm thick. Ornamentation granulate (LM), microechinate-perforate (SEM).

Distribution: North China (Fig. 5f), Iran, Afghanistan and Kazakhstan. Habitat: Sand dunes.

*Iljinia regelii* (Bunge) Korovin (Plate 8j-l)

Pollen grains spheroidal, 12.5 (11.1-13.9) μm × 12.1 (10.7-13.4) μm, small size. P/E ratio 1.03 (1.00-1.06). Apertures pantoporate with 16 (10-24) pores, pores circular approximately 4.1 (3.6-4.8) μm in diameter evenly distributed on the surface of grains, echinate with granules. Exine ca. 1.4 (1.2-1.8) μm thick. Ornamentation granulate (LM), microechinate-perforate (SEM).

Distribution: Northwest China (Fig. 5g), Mongolia and Kazakhstan. Habitats: Gobi, alluvial fans, gravel dunes and dry slopes.

*Sympegma regelii* Bunge (Plate 8m-o)

Pollen grains spheroidal, 18.5 (15.7-21.9) μm × 17.6 (15.1-21.4) μm, small size. P/E ratio 1.05 (1.02-1.17). Apertures pantoporate with 22 (18-28) pores, pores circular approximately 2.4 (1.9-3.1) μm in diameter evenly distributed on the surface of grains, echinate with granules. Exine ca. 1.8 (1.3-2.3) μm thick. Ornamentation granulate (LM), microechinate-perforate (SEM).

Distribution: Northwest China (Fig. 5h), Kazakhstan and Mongolia. Habitats: Slightly saline-alkaline deserts, dry hillsides, alluvial fans and edges of canals.

*Kalidium schrenkianum* Bunge ex Ung.-Sternb. (Plate 9a-c)

Pollen grains spheroidal, 19.0 (16.7-22.1) μm × 18.2 (15.8-21.5) μm, small size. P/E ratio 1.05 (1.00-1.12). Apertures pantoporate with 24 (18-30) pores, pores circular approximately 2.4 (1.9-2.9) μm in diameter evenly distributed on the surface of grains, echinate with granules. Exine ca. 2.0 (1.6-2.3) μm thick. Ornamentation granulate (LM), microechinate-perforate (SEM).

Distribution: Xinjiang of China (Fig. 6a) and Central Asia. Habitats: Saline-alkaline lands, saline lakesides.

*Kalidium cuspidatum* (Ung.-Sternb.) Grubov (Plate 9d-f)

Pollen grains spheroidal, 17.4 (14.1-19.6) μm × 16.5 (12.6-18.8) μm, small size. P/E ratio 1.05 (1.00-1.13). Apertures pantoporate with 18 (16-24) pores, pores circular approximately 2.1 (1.2-2.8) μm in diameter evenly distributed on the surface of grains, echinate with granules. Exine ca. 1.8 (1.5-2.1) μm thick. Ornamentation granulate (LM), microechinate-perforate (SEM).

Distribution: West and north of China (Fig. 6b) and Mongolia. Habitats: Edge of saline lakes and saline-alkaline lands.

*Krascheninnikovia ceratoides* (L.) Gueldenst. (Plate 9g-i)

Pollen grains spheroidal, 23.8 (20.3-27.2) μm × 22.6 (20.4-25) μm, small size. P/E ratio 1.05 (1.01-1.20). Apertures pantoporate with 42 (34-50) pores, pores circular approximately 2.4 (1.3-3.3) μm in diameter evenly distributed on the surface of grains, echinate with granules. Exine ca. 2.3 (2.0-2.6) μm thick. Ornamentation granulate (LM), microechinate-perforate (SEM).

Distribution: Northwestern China (Fig. 6c) and arid region of Eurasia. Habitats: Edge of saline lakes and saline-alkaline lands.

*Krascheninnikovia compacta* (Losinsk.) Grubov (Plate 9j-l)

Pollen grains spheroidal, 24.6 (19.2-25.7) μm × 23.4 (18.6-25.5) μm, small size. P/E ratio 1.05 (1.00-1.11). Apertures pantoporate with 38 (30-44) pores, pores circular approximately 2.0 (1.1-2.6) μm in diameter evenly distributed on the surface of grains, echinate with granules. Exine ca. 2.2 (1.6-2.5) μm thick. Ornamentation granulate (LM), microechinate-perforate (SEM).

Distribution: Northwest China (Fig. 6d) and eastern Pamir. Habitats: Mountain slopes and gravelly areas (3500-5000 m).

*Nanophyton erinaceum* (Pall.) Bunge (Plate 9m-o)

Pollen grains spheroidal, 18.6 (16.4-19.8) μm × 18.0 (16-19.2) μm, small size. P/E ratio 1.03 (1.00-1.07). Apertures pantoporate with 16 (14-20) pores, pores circular approximately 3.2 (2.5-3.8) μm in diameter evenly distributed on the surface of grains, echinate with granules. Exine ca. 1.8 (1.5-2.3) μm thick. Ornamentation granulate (LM), microechinate-perforate (SEM).

Distribution: Xinjiang of China (Fig. 6e), Mongolia, Kazakhstan and Siberia. Habitats: Gobi, stony slopes and dry soil areas.

*Salsola passerina* Bunge (Plate 10a-c)

Pollen grains spheroidal, 24.9 (21-27.2) μm × 24.2 (20.7-26.7) μm, small size. P/E ratio 1.03 (1.01-1.18). Apertures pantoporate with 18 (12-22) pores, pores circular approximately 4.5 (3.9-5.0) μm in diameter evenly distributed on the surface of grains, echinate with granules. Exine ca. 2.3 (1.8-3.0) μm thick. Ornamentation granulate (LM), microechinate-perforate (SEM).

Distribution: Northwest China (Fig. 6f) and Mongolia. Habitats: Mountain slopes, gravelly beaches.

*Salsola abrotanoides* Bunge (Plate 10d-f)

Pollen grains spheroidal, 19.0 (17.2-20.8) μm × 18.3 (16.9-20.1) μm, small size. P/E ratio 1.04 (1.00-1.10). Apertures pantoporate with 18 (14-22) pores, pores circular approximately 2.9 (2-3.5) μm in diameter evenly distributed on the surface of grains, echinate with granules. Exine ca. 1.9 (1.5-2.2) μm thick. Ornamentation granulate (LM), microechinate-perforate (SEM).

Distribution: Northwest China (Fig. 6g) and Mongolia. Habitats: Mountain slopes, piedmont alluvial fans and gravelly beaches.

*Suaeda physophora* Pall. (Plate 10g-i)

Pollen grains spheroidal, 18.8 (17.3-21.8) μm × 17.5 (14.8-20.6) μm, small size. P/E ratio 1.07 (1.00-1.18). Apertures pantoporate with 36 (30-44) pores, pores circular approximately 1.2 (0.9-1.5) μm in diameter evenly distributed on the surface of grains, echinate with granules. Exine ca. 1.9 (1.5-2.2) μm thick. Ornamentation granulate (LM), microechinate-perforate (SEM).

Distribution: Northwestern China (Fig. 6h), eastern Europe, Central Asia and western Siberia. Habitats: Gobi and saline-alkaline and dry hillsides.

*Suaeda microphylla* Pall. (Plate 10j-l)

Pollen grains spheroidal, 23.3 (20.0-27.5) μm × 22.4 (17.7-26.7) μm, small size. P/E ratio 1.04 (1.00-1.14). Apertures pantoporate with 38 (32-46) pores, pores circular approximately 1.1 (1.0-1.4) μm in diameter evenly distributed on the surface of grains, echinate with granules. Exine ca. 2.2 (1.8-2.7) μm thick. Ornamentation granulate (LM), microechinate-perforate (SEM).

Distribution: Northwest China (Fig. 7a), Central Asia and Caucasus. Habitats: Gobi, sand dunes, lakesides and saline-alkaline deserts.

**Caryophyllaceae**

*Gymnocarpos przewalskii* Bunge ex Maxim. (Plate 10m-o)

Pollen grains spheroidal, 23.6 (18.4-27.8) μm × 22.7 (17.1-26.5) μm, small size. P/E ratio 1.04 (1.01-1.09). Apertures pantoporate with 14 (10-18) pores, pores with annulus circular approximately 1.1 (1.0-1.4) μm in diameter evenly distributed on the surface of grains, echinate with granules. Exine ca. 2.2 (1.6-2.9) μm thick. Ornamentation granulate (LM), microechinate-perforate (SEM).

Distribution: Northwestern China (Fig. 7b) and Mongolia. Habitats: Dry pools, Gobi and gravel slopes in desert areas (1000-2500 m).

**Tamaricaceae**

*Reaumuria soongarica* (Pall.) Maxim. (Plate 11a-c)

Pollen grains subprolate, 13.1 (11.5-14.9) μm × 10.4 (9-12.1) μm, small size. P/E ratio 1.27 (1.15-1.47). Elliptical in equatorial view and trilobate circular in polar view. Apertures tricolpate. Colpi long, nearly reaching the poles, sunken, broad, margin regular. Exine ca. 1.2 (1.1-1.4) μm thick. Ornamentation psilate (LM), reticulate (SEM). Muri smooth. Lumina small.

Distribution: Northwestern China (Fig. 7c) and Mongolia. Habitats: Piedmont alluvium, alluvial plains and Gobi erosion surfaces on the edges of the lowlands and matrix for coarse gravel Gobi in desert regions.

*Tamarix chinensis* Lour. (Plate 11d-f)

Pollen grains spheroidal, 16.3 (13.2-20.7) μm × 15.5 (14-18.4) μm, small size. P/E ratio 1.05 (0.87-1.28). Elliptical in equatorial view and trilobate circular in polar view. Apertures tricolpate. Colpi long, nearly reaching the poles, sunken, broad, margin regular. Exine ca. 1.6 (1.2-1.9) μm thick. Ornamentation psilate (LM), reticulate (SEM). Muri smooth. Lumina large and uniform.

Distribution: North and east of China (Fig. 7d), Japan and the United States. Habitats: River alluvial plains, beaches, moist saline land and sandy lands.

**Poaceae**

*Psammochloa villosa* (Trin.) Bor (Plate 11g-i)

Pollen grains subprolate, 43.1 (40.9-46.8) μm × 35.2 (31.3-42.5) μm, medium size. P/E ratio 1.23 (1.05-1.39). Aperture ulcus, pore circular approximately 3.4 (3.2-3.7) μm in diameter and covered by membrane with granulae. Annulus around the pore approximately 8.5 (7-10.7) μm in diameter. Exine ca. 2.3 (2.1-2.8) μm thick. Ornamentation psilate (LM), microgranulate (SEM).

Distribution: Northwest China (Fig. 7e) and Mongolia. Habitat: Sand dunes (910-2900 m).

**Salicaceae**

*Populus euphratica* Oliv. (Plate 11j-l)

Pollen grains spheroidal, 26.8 (19.3-37.2) μm × 25.0 (19.1-36.4) μm, medium size. P/E ratio 1.07 (1.01-1.2). Inaperturate, exine ca. 1.6 (1.2-2.1) μm thick. Ornamentation microgranulate (LM), granulate (SEM).

Distribution: Northwestern China (Fig. 7f), Mongolia, Central Asia, Caucasus, Egypt, Syria, India, Iran, Afghanistan and Pakistan. Habitats: Basins, valleys and plains.

**Convolvulaceae**

*Convolvulus tragacanthoides* Turcz. (Plate 11m-o)

Pollen grains subprolate, 61.9 (51.7-78.4) μm × 47.5 (34.5-63.8) μm, large size. P/E ratio 1.33 (0.95-1.59). Elliptical in equatorial view and trilobate circular in polar view. Apertures tricolpate. Colpi long, nearly reaching the poles, wide, echinate with granules. Exine ca. 5.5 (4.7-7.0) μm thick. Ornamentation microgranulate (LM), granulate-reticulate (SEM). Lumina heterobrochate.

Distribution: Northwestern and northern China (Fig. 7g), Mongolia and Central Asia. Habitats: Stony ground and Gobi.

**Ephedraceae**

*Ephedra przewalskii* Stapf (Plate 11p-r)

Pollen grains prolate, 29.9 (24.5-34.2) μm × 16.3 (14.8-18.2) μm, medium size. P/E ratio 1.84 (1.44-2.20). Elliptical in equatorial view. Inaperturate. Exine ca. 2.0 (1.8-2.4) μm thick. Ornamentation striate (LM), fossulate (SEM).

Distribution: Northwestern China (Fig. 7h) and Mongolia. Habitats: Dry desert and arid foothills, saline-gravelly land.
